# *TCF4*-mediated Fuchs endothelial corneal dystrophy: Insights into a common trinucleotide repeat-associated disease

**DOI:** 10.1016/j.preteyeres.2020.100883

**Published:** 2021-03

**Authors:** Michael P. Fautsch, Eric D. Wieben, Keith H. Baratz, Nihar Bhattacharyya, Amanda N. Sadan, Nathaniel J. Hafford-Tear, Stephen J. Tuft, Alice E. Davidson

**Affiliations:** a Department of Ophthalmology, 200 1st St SW, Mayo Clinic, Rochester, MN, 55905, USA; b Department of Biochemistry and Molecular Biology, 200 1st St SW, Mayo Clinic, Rochester, MN, USA; c University College London Institute of Ophthalmology, London, ECIV 9EL, UK; d Moorfields Eye Hospital, London, EC1V 2PD, UK

**Keywords:** Fuchs endothelial corneal dystrophy, Repeat-expansion, Transcription factor 4, Triplet repeat-mediated disease, Trinucleotide repeat, FECD, CTG18.1, RNA toxicity, RAN translation

## Abstract

Fuchs endothelial corneal dystrophy (FECD) is a common cause for heritable visual loss in the elderly. Since the first description of an association between FECD and common polymorphisms situated within the transcription factor 4 (*TCF4*) gene, genetic and molecular studies have implicated an intronic CTG trinucleotide repeat (CTG18.1) expansion as a causal variant in the majority of FECD patients. To date, several non-mutually exclusive mechanisms have been proposed that drive and/or exacerbate the onset of disease. These mechanisms include (i) *TCF4* dysregulation; (ii) toxic gain-of-function from *TCF4* repeat-containing RNA; (iii) toxic gain-of-function from repeat-associated non-AUG dependent (RAN) translation; and (iv) somatic instability of CTG18.1. However, the relative contribution of these proposed mechanisms in disease pathogenesis is currently unknown. In this review, we summarise research implicating the repeat expansion in disease pathogenesis, define the phenotype-genotype correlations between FECD and CTG18.1 expansion, and provide an update on research tools that are available to study FECD as a trinucleotide repeat expansion disease. Furthermore, ongoing international research efforts to develop novel CTG18.1 expansion-mediated FECD therapeutics are highlighted and we provide a forward-thinking perspective on key unanswered questions that remain in the field.

## Highlights

• FECD is a common, age-related corneal dystrophy.
• The majority of cases are associated with expansion of a CTG repeat (CTG18.1).
• FECD is the most common trinucleotide repeat expansion disease in humans.
• Evidence supports multiple molecular mechanisms underlying the pathophysiology.
• Novel CTG18.1-targeted therapeutics are in development.

## 1 Introduction

### 1.1 Fuchs endothelial corneal dystrophy

**Fig. 1:**
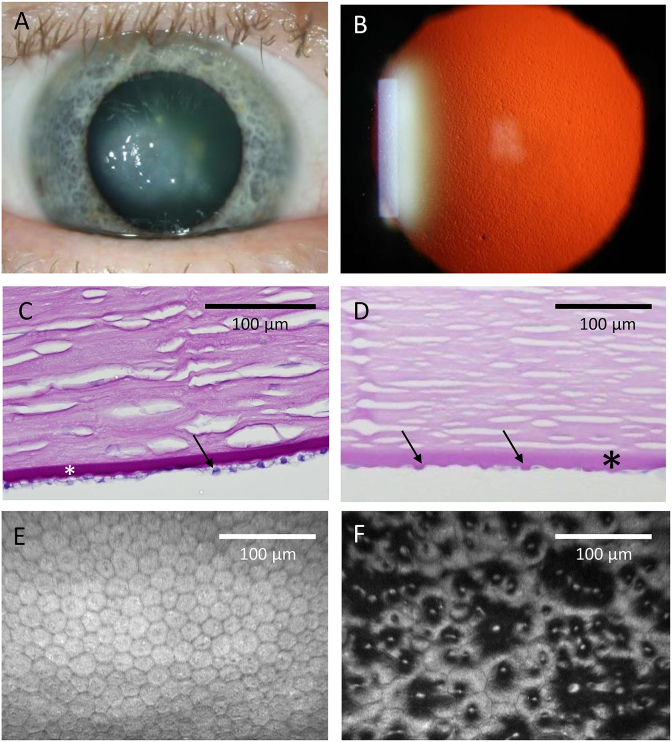
**Clinical images of Fuchs endothelial corneal dystrophy (FECD).** A. Edema of the central cornea due to FECD. B. Retro-illumination photograph of a cornea with FECD showing numerous guttae that give a stippled appearance. C. Periodic acid-Schiff (PAS)-stained cross-section of normal cornea (endothelial cells, arrow; Descemet's membrane, asterisk). D. PAS-stained cross-section of cornea with FECD showing posterior elevations (guttae, arrows) arising from the thickened Descemet membrane (asterisk). E. *In vivo* confocal image of normal corneal endothelial cells show a regular hexagonal pattern. F. *In vivo* confocal image of FECD corneal endothelium with disrupted hexagonal cell pattern. Dark areas represent guttae.

Fuchs endothelial corneal dystrophy (FECD) is a common bilateral eye disease and a frequent cause of cornea-related blindness. The defining clinical sign of early disease is the presence of microscopic collagenous excrescences of corneal endothelial basement (Descemet) membrane (referred to as guttae) which protrude posteriorly toward the anterior chamber (Fig. 1). FECD is usually diagnosed in the fourth decade of life or later, though guttae can be present for many years before noticeable symptoms develop (Goar, 1933). As individuals age, the optical quality of the cornea degrades as the number of guttae increase, resulting in reduced contrast sensitivity, increased glare and loss of visual acuity (Baratz et al., 2012; Patel et al., 2012; van der Meulen et al., 2011). With more advanced disease, stromal and epithelial edema ensues along with subepithelial fibrosis which can result in pain and severe vision loss.

### 1.2 Characteristics of corneal endothelial cells in FECD

The main function of corneal endothelial cells is to maintain corneal deturgescence by removing fluid from the stroma via specialized ion transporters dependent on Na^+^/K^+^-ATPase activity (Bonanno, 2003, 2012; Li et al., 2016). As FECD progresses with age, the number of guttae increases and endothelial cell density decreases, especially in the central cornea, resulting in the loss of the regular hexagonal cellular array (Fig. 1). The corneal endothelium is vulnerable to endothelial cell loss given that the cells in vivo are arrested in the G_1_ phase of the cell cycle and therefore have minimal proliferation potential (Joyce, 2012). Consequently, the loss of endothelial cells is often associated with a failure of the endothelium to effectively maintain the corneal aqueous barrier and deturgescence, resulting in corneal edema, diminished clarity, and vision loss.

### 1.3 Epidemiology of FECD

The recorded incidence and prevalence of FECD varies widely over different ethnic groups. In the United States and Europe, the prevalence of the disease is estimated to be 4–5% among persons over the age of 40 years, while FECD is considered uncommon in Japan, Saudi Arabia and in Chinese Singaporeans (Eghrari and Gottsch, 2010; Krachmer et al., 1978; Lorenzetti et al., 1967; Santo et al., 1995; Vithana et al., 2008). When assessed with specular microscopy, the prevalence of guttae is higher in white (11.2%) than East Asian (5.5%) populations and higher in females (5.5%–11%) than males (1.5%–7%) (Kitagawa et al., 2002; Zoega et al., 2006). Females are affected up to three times more often than males in several different ethnic populations while also having more severe disease (Afshari et al., 2006; Higa et al., 2011; Kitagawa et al., 2002; Zoega et al., 2006). Corneal transplantation is currently the only treatment for advanced FECD (Matthaei et al., 2017) accounting for 39% of United States primary corneal transplant cases in 2018 (Eye-Bank, 2019).

## 2 Genetic association of *TCF4* and FECD

The familial nature of FECD through an autosomal dominant mode of transmission was first described in 1971 (Cross et al., 1971). Several years later, a confirmatory study described 64 families also with an inheritance pattern consistent with an autosomal dominant trait (Krachmer et al., 1978). This cohort showed variable penetrance and expression and a female preponderance. Since this landmark study, application of traditional linkage and candidate gene screening approaches have identified rare and presumed disease-associated variants in several genes including *AGBL1*, *COL8A2, LOXHD1, SLC4A11 and ZEB1* (Table 1) (Afshari et al., 2017; Biswas et al., 2001; Gupta et al., 2015; Mehta et al., 2008; Riazuddin et al., 2010a, 2010b, 2012, 2013). Despite these findings, presumed disease-causing mutations in these genes collectively account for only a small proportion of total FECD. Recently, a large multi-centre genome wide association study (GWAS) in the United States analysing 2075 FECD patients and 3342 control subjects identified common polymorphisms in proximity to *KANK4*, *LAMC1* and *LINC00970/ATP1BP1*, in addition to *TCF4* (discussed below), that showed a significant association with increased FECD risk (Afshari et al., 2017). Notably, none of the associated loci identified by the Afshari et al. GWAS encompass or were found to be in close proximity to the aforementioned genes (*AGBL1*, *COL8A2*, *LOXHD1*, *SLC4A11* and *ZEB1*) hypothesised to harbour rare disease-associated variants. Pathogenic mechanisms underlying these disease-causing and disease-associated genes are not the focus of this review, and additional information regarding their FECD-association has recently been described elsewhere (Zhang et al., 2019, Ong Tone et al., 2020).

**Table 1:** **Genes and loci associated with Fuchs endothelial corneal dystrophy (FECD)**.

Associated gene or loci	Protein	OMIM	Genomic coordinates (GRCh38)	Most significantly associated SNP	Reference
**Gene harbouring presumed causative variant(s)**					
*TCF4*	Transcription factor 4	613267	18:55,222,184–55,635,956	rs613872 rs784257	Baratz et al. (2010); Wieben et al. (2012) Afshari et al. (2017)
*COL8A2*	Collagen Type VIII Alpha 2 Chain	136800	1:36,095,238–36,126,206	NA	Biswas et al. (2001)
*SLC4A11*	Solute carrier family 4 (sodium borate cotransporter), member 11	613268	20:3,227,416–3,241,483	NA	Vithana et al. (2008)
*ZEB1*	Zinc finger E box-binding homeobox 1	613270	10:31,318,416–31,529,813	NA	Mehta et al. (2008)
*AGBL1*	ATP/GTP-binding protein-like 1	615523	15:86,079,619–87,031,475	NA	Riazuddin et al. (2013)
*LOXHD1*	Lipoxygenase homology domain-containing 1	NA	18:46,476,960–46,657,114	NA	Riazuddin et al. (2012)
**Associated loci identified via GWAS**					
*KANK4*	KN motif- and ankyrin repeat domain-containing protein 4	NA	1:62,236,164–62,319,433	rs79742895	Afshari et al. (2017)
*LAMC1*	Laminin, gamma-1	NA	1:183,023,419–183,145,591	rs3768617	Afshari et al. (2017)
*LINC00970/ATP1B1*	ATPase, Na+/K+ transporting, beta-1 polypeptide	NA	1:169,106,689–169,132,718	rs1200114	Afshari et al. (2017)

OMIM, online inheritance in man; GWAS, genome wide association study; SNP, single nucleotide polymorphism.

### 2.1 Common TCF4 polymorphisms are associated with FECD

**Fig. 2:**
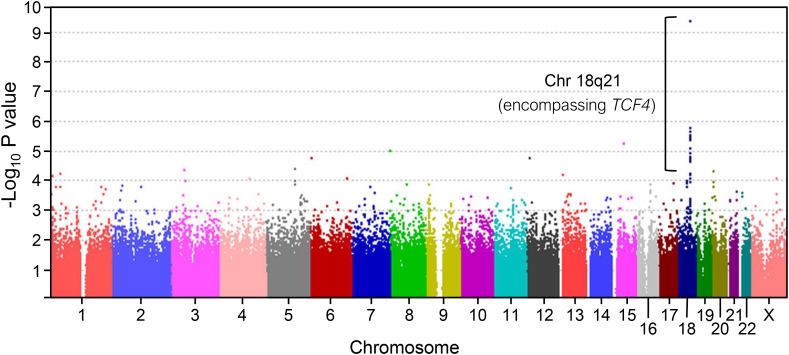
**Manhattan plot illustrating a genome wide significant association between Fuchsendothelialcornealdystrophy (FECD) and a region on chromosome 18, encompassing *TCF4*.** Genome wide association study (GWAS) comparing 338,727 Single-Nucleotide Polymorphisms (SNPs) and FECD. The negative log of the P values of association between genotyped SNPs and an FECD discovery cohort is plotted against chromosomal location. One region on chromosome 18, encompassing *TCF4*, was found to reach genome wide significance (p = 1.0 × 10^−12^) within this discovery cohort. Used with permission from Baratz et al. (2010).

In 2010, we performed a GWAS using a North American Caucasian cohort of 130 FECD patients and 260 controls (Fig. 2) (Baratz et al., 2010). Despite the relatively small cohort size, several non-coding single-nucleotide polymorphisms (SNPs; rs613872, rs17595731, rs9954153, rs2286812) on chromosome 18 achieved genome-wide significance (P = 5 × 10^−8^) with rs613872 showing the strongest association (P = 1.0 × 10^−12^). We confirmed this association in an independent replication cohort comprising an additional 150 affected and 150 control subjects (P = 1.79 × 10^−13^). These SNPs were found to cluster within and around the transcription factor 4 (*TCF4)* gene (chromosome 18q21.2; OMIM # 602272; ENSG00000196628). In the combined experimental and replication cohorts, one copy of the rs613872 minor allele (heterozygotes, GT) conferred a 5.5 fold risk of FECD and two copies of the minor allele (homozygotes, GG) conferred a 30-fold risk of FECD. Notably, an earlier FECD familial linkage study in 2006 had previously identified a disease-associated region on chromosome 18q21 encompassing *TCF4* (Sundin et al., 2006). However, the unusually high odds ratios obtained from our GWAS (Baratz et al., 2010) provided the first evidence to suggest that a true causal variant could be directly associated with rs613872 and underlie disease in a large proportion of total cases (Wright and Dhillon, 2010).

The association of FECD with common SNPs encompassed within and around *TCF4* has since been replicated by several independent groups in ethnically diverse patient populations (Li et al., 2011; Riazuddin et al., 2011; Thalamuthu et al., 2011). Additionally, the recent multi-centre GWAS further confirmed the association between common variants in *TCF4* and the disease (Afshari et al., 2017). In this study, a SNP situated in an intergenic region upstream of *TCF4* (rs784257) conferred the most significant association (P = 2.5 × 10^−200^).

### 2.2 TCF4 contains a non-coding trinucleotide repeat (TNR) expansion

Mutations and common polymorphisms within *TCF4* have been implicated in several diseases including bipolar disorder, schizophrenia, Pitt-Hopkins Syndrome (OMIM # 610954), and Sonic Hedgehog (SHH) medulloblastoma tumours (Blake et al., 2010; Blanluet et al., 2019; Forrest et al., 2012, 2018; Hellwig et al., 2019; Rannals and Maher, 2017; Roussos, 2012; Sweatt, 2013; Xia et al., 2018). Due to molecular interrogation of a locus on chromosome 18 and its association with bipolar disorder, a CTG trinucleotide repeat was discovered in *TCF4* and subsequently named CTG18.1 (see Fig. 4A and B) (Breschel et al., 1997). Despite not identifying a disease association between CTG18.1 and bipolar disorder, Breschel and co-workers did discover expanded copies of the repeat in both bipolar and control cohorts at frequencies of ~3% (Table 2). Intriguingly, this approximately corresponds to the prevalence of FECD in the northern European population now established to be attributed to CTG18.1 expansion. Furthermore, these authors observed stable inheritance of unexpanded allele lengths, whereas moderate (53–250 repeats) and large (800–2100 repeats) expansions were reported to be unstably inherited during parent to child transmission (Breschel et al., 1997). A subsequent study utilizing bipolar disorder, schizophrenia, affective disorder and non-specific ataxia cohorts later confirmed the lack of associations between CTG18.1 expansion and these diseases (Breschel et al., 1997; McInnis et al., 2000). While no clear genetic association between CTG18.1 repeat length and psychiatric disorders have been identified since, genetic expansions of repetitive regions elsewhere in the genome have been linked to over 40 diseases [reviewed in (Paulson, 2018; Rodriguez and Todd, 2019)]. These repeat expansion diseases are typically progressive neuro- and neuro-muscular degenerations such as fragile X syndrome, Huntington's disease, *C9ORF72*-associated amyotrophic lateral sclerosis and frontotemporal dementia (C9ORF72 ALS/FTD), and myotonic dystrophy type 1 (DM1) and type 2 (DM2) (Brook et al., 1992; DeJesus-Hernandez et al., 2011; Group, 1993; Kremer et al., 1991; Liquori et al., 2001; Orr and Zoghbi, 2007; Paulson, 2018; Renton et al., 2011).

**Table 2:** **Summary of CTG18.1 genotyping studies performed across multi-ethnic Fuchs endothelial corneal dystrophy (FECD) patient and control cohorts**.

Ethnicity, as reported in original study	FECD cases with CTG18.1 expansion (%)	Controls with CTG18.1 expansion (%)	Refereance
British Caucasian	77.3%^†^	4.2%^†^	Zarouchlioti et al. (2018)
Czech Republic	81.1%^†^	–	Zarouchlioti et al. (2018)
American	79%^†^ 73%* 62%* 63%*	3%^†^ 7%* 3.6%*	Wieben et al. (2012); Mootha et al. (2014); Vasanth et al. (2015); Eghari et al. (2017a)
German	77^†^; 79%^†^ 79%^†^	10.8^†^ 11.5%^†^	Foja et al. (2017); Okumura et al. (2019a); Luther et al. (2016)
Russian	72%*	5%*	Skorodumova et al. (2018)
Belgian	–	8%*	Del-Favero et al. (2002)
Swedish	–	3%*	Del-Favero et al. (2002)
Croatian	–	6%*	Del-Favero et al. (2002)
Danish	–	3%*	Del-Favero et al. (2002)
Scottish	–	7%*	Del-Favero et al. (2002)
Northern European	–	3%^†^	Breschel et al. (1997)
Australian	51%*	5%*	Kuot et al. (2017)
Thai	39%*	0%*	Okumura et al. (2019c)
Singaporean Chinese	44%*	1.7%*	Xing et al. (2014)
Japanese	26%^†^	0%^†^	Nakano et al. (2015)
Indian	17%^†^	3%^†^	Rao et al. (2017)
Inidan (Odisha and West Bengal)	34%^†^	5%^†^	Nanda et al. (2014)
African American	35%*	–	Eghari et al. (2017a)

*, >40 repeats used as criteria for expansion; ^†^, >50 repeats used as criteria for expansion; -- not screened.

### 2.3 FECD is associated with a trinucleotide repeat expansion in the non-coding region of TCF4

Lack of disease-associated mutations within the coding regions of *TCF4* reported by us and subsequently confirmed by Riazuddin et al. (Baratz et al., 2010; Riazuddin et al., 2011)*,* raised the possibility that the genetic variant(s) underlying the FECD association could reside in a non-coding region of *TCF4*. Given the natural history of FECD as an autosomal dominant, late-onset genetic disease, we explored the possibility that variations in CTG18.1 repeat length could represent the underlying causal variant. In 2012, we reported CTG18.1 genotyping results for 66 Caucasians with a confirmed diagnosis of FECD (Wieben et al., 2012). Remarkably, 52/66 (79%) had repeat lengths ≥50, with three individuals harbouring a CTG expansion >1500. This finding was in stark contrast to the concurrently investigated control population of which only 2/63 individuals (3%) with FECD negative corneas had ≥50 copies of the CTG repeat. Furthermore, our data demonstrated that the presence of one or more expanded (≥50 copies) CTG18.1 alleles was more specific in predicting FECD than any previously described FECD-associated variant (Wieben et al., 2012).

The association between CTG18.1 repeat length and FECD has now been replicated within numerous multi-ethnic cohorts (Table 2). Regardless of subtle variability in designated expansion thresholds used by ourselves and others (ranging from ≥40 to ≥50 repeats), the highest frequencies of CTG18.1 expansions have consistently been reported in predominantly Caucasian FECD populations including those in the United States (62–79%) (Eghrari et al., 2017a; Mootha et al., 2014; Vasanth et al., 2015; Wieben et al., 2012), Germany (77%–79%) (Foja et al., 2017; Okumura et al., 2019a), United Kingdom (77%) (Zarouchlioti et al., 2018), Czech Republic (81%) (Zarouchlioti et al., 2018), and Russia (72%) (Skorodumova et al., 2018). A lower prevalence was noted in a cohort of Australian FECD patients (51%), but the experimental methods employed in this study were unable to detect large expansions which may have decreased ascertainment rates (Kuot et al., 2017). Interestingly, the correlation between CTG18.1 expansion and FECD is also striking, although typically lower, in other non-Caucasian ethnic groups investigated to date. This includes African Americans (35%) (Eghrari et al., 2017a), Indians (17% and 34%) (Nanda et al., 2014; Rao et al., 2017), Japanese (26%) (Nakano et al., 2015), Singaporean Chinese (44%) (Xing et al., 2014), and Thai (39%) (Okumura et al., 2019c). Notably, CTG18.1 genotyping studies have consistently identified a bimodal distribution of repeat lengths, with the vast majority of patients typically harbouring repeat sizes of <30 or >50 (Zarouchlioti et al., 2018).

### 2.4 Challenges and limitations associated with screening the CTG18.1 repeat

The diagnosis and prognosis of repeat expansion is often dependent on accurate sizing of mutant alleles (Paulson, 2018). Expanded repeats remain intractable to short-read next-generation sequencing technologies due to their innate repetitive nature, size, and typically high GC content (Ardui et al., 2018; Treangen and Salzberg, 2011). These regions are usually investigated in a diagnostic setting using PCR-based amplification methods and Southern blotting (Gomes-Pereira and Monckton, 2004; Kohwi, 2004; Mootha et al., 2014; Warner et al., 1996; Wieben et al., 2012). Although these techniques provide an inexpensive and relatively simple method of estimating pathogenic repeat lengths, they all lack the ability to accurately determine repeat size and fail to provide sequence level resolution (Table 3).

**Table 3:** **Screening methods employed to measure trinucleotide repeat length.** Relative advantages and disadvantages of methods used to measure repeat length and instability.

	Southern blot	STR analysis	SP-PCR	TP-PCR	No-Amp
PCR dependent		✓	✓	✓	
High total DNA input requirement	✓				✓
Repeat size estimate	✓	✓	✓	✓	
Labour intensive	✓		✓		
Detection of >130 repeats	✓		✓	✓*	✓
Sequence level resolution					✓
Detection of biallelic expansions	✓	✓**	✓	✓*	✓
Detection of allele level instability			✓		✓

STR, Short tandem repeat; SP-PCR, Small pool-polymerase chain reaction; TP-PCR, Triplet repeat primed-polymerase chain reaction; *, Only allows presence or absence detection at >130 repeats; **, Only if both alleles are <130 repeat.

To solve this problem, novel amplification-free sequencing methods have been developed that involve the use of targeted DNA enrichment by CRISPR/Cas9 in conjunction with sequencing of enriched native DNA by long-read single molecule sequencing platforms (e.g. Pacific Biosciences single molecule real time (SMRT) and Oxford Nanopore Technology (ONT) sequencing) (Ebbert et al., 2018; Gabrieli et al., 2018; Hoijer et al., 2018; Pham et al., 2016; Tsai et al., 2017). Such methods avoid the introduction of PCR-artefactual variation and, furthermore, enable sequence level resolution of single DNA molecules encompassing repetitive regions, such as CTG18.1. We have recently used Pacific Biosciences ‘No-Amp’ method which combines amplification-free enrichment with SMRT sequencing to sequence CTG18.1 in FECD patient cohorts (see Fig. 1 in Hafford-Tear et al., 2019 for a schematic summary of the methodology applied) (Hafford-Tear et al., 2019). These data have demonstrated that the ‘No Amp’ method enables accurate sizing and sequence level resolution of CTG18.1 alleles. Furthermore, the method revealed that expanded copies of CTG18.1 behave dynamically and are highly unstable, both contracting and expanding in length within peripheral blood leukocytes (Hafford-Tear et al., 2019; Wieben et al., 2019a). The biological relevance of this finding is discussed in Section 3.4. Challenges remain for this technology in improving target enrichment specificity, reducing DNA input requirements and developing improved informatics for sequence analysis (Hoijer et al., 2018, 2020; Jinek et al., 2012; Wieben et al., 2019a). The retrieval of high molecular weight DNA from sample material also remains a limiting factor for all of the above screening methodologies (Table 3).

## 3 Evidence of trinucleotide repeat expansion disease mechanisms underlying FECD

**Fig. 3:**
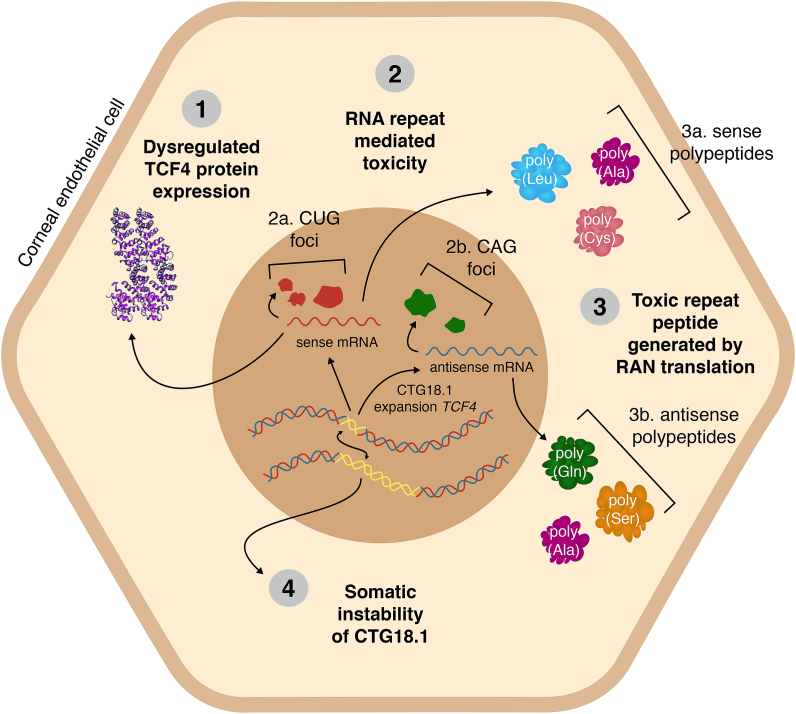
**Mechanisms of cellular dysregulation associated with CTG18.1 expansions.** Four non-mutually exclusive mechanisms have been proposed to drive and/or exacerbate the onset of CTG18.1 expansion-mediated FECD, including; (1) dysregulated expression of *TCF4* transcripts, (2) accumulation of toxic (a) sense (CUG)_n_ and (b) antisense-derived (CAG)_n_ repetitive RNA transcripts, (3) RAN translation of repetitive RNA transcripts, and (4) age and tissue-dependant somatic instability of the repeat element.

The discovery that non-coding CTG18.1 repeat expansions are detected in the vast majority of FECD cases has revolutionized our understanding of the disease (Wieben et al., 2012). Since the original finding was published in 2012, substantial progress has been made towards unravelling the pathophysiology of this common, age-related repeat expansion disease. Researchers have drawn inspiration from the repeat expansion research community and four distinct, non-mutually exclusive disease mechanisms have been hypothesised to drive and/or exacerbate the onset of disease (summarized in Fig. 3). These include the potential dysregulation of *TCF4* expression, RNA-induced toxicity, Repeat Associated Non-AUG (RAN) translation and the instability of CTG18.1 repeat length in affected tissue. Herein we discuss the evidence in support of each of these distinct mechanisms and highlight current uncertainties and challenges that remain.

**Fig. 4:**
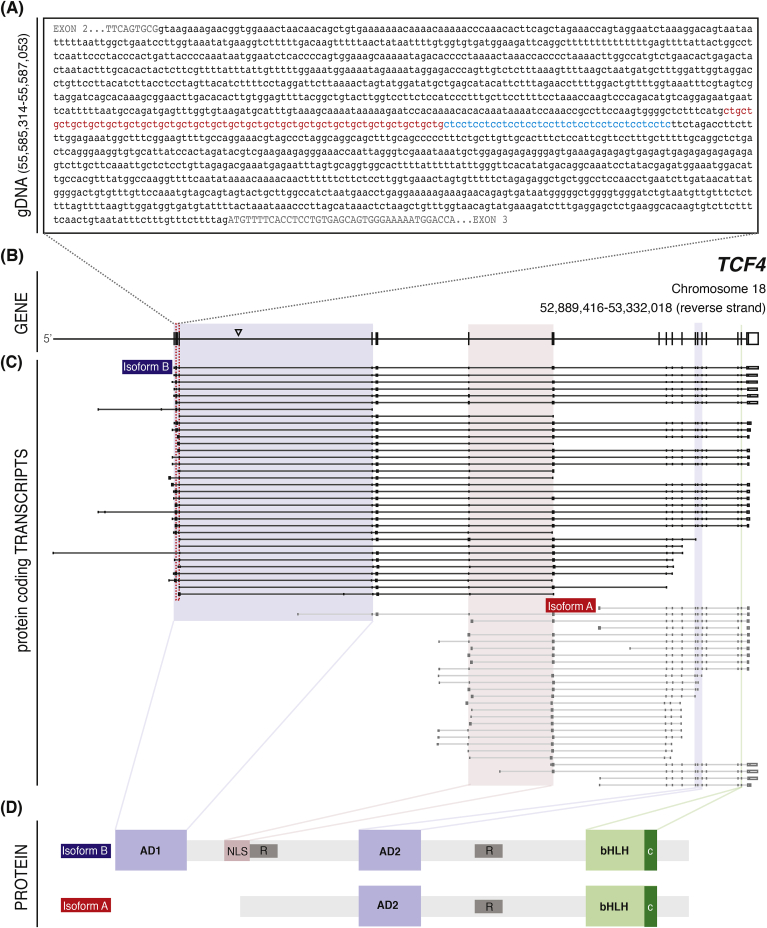
**Schematic illustration of *TCF4* genomic sequence encompassing CTG18.1, defined protein coding TCF4 isoforms, and characterized TCF4 functional domains.** A. Genomic sequence surrounding the CTG18.1 repeat element (red) and adjacent CTC repeat (blue). Intronic sequence surrounding the repeat is shown in lowercase black lettering. Exonic sequence is represented by uppercase letters. B. A schematic of the canonical *TCF4* transcript (ENST00000354452.8; NM_001083962.2) encoding Isoform B (ENSP00000346440.3). Arrowhead shows the relative position of the SNP rs613872. Sequence is presented in a 5′ to 3′ orientation. C. A schematic representation of all characterized, protein coding, *TCF4* transcripts reported in Ensembl. Transcripts that contain or are in close proximity to the CTG18.1 repeat (red dotted box) are grouped and colored in black. Those transcripts which do not encompass the repeat are colored grey. The transcripts encoding isoform A (TCF4-204 – red) and Isoform B (TCF4-201 - blue) are labelled. Colored boxes denote exons which code for specific TCF4 functional domains. D. Schematic of TCF4 protein functional domains characterized within isoform A and isoform B. Activation domain 1 (AD1) and activation domain 2 (AD2) are represented in blue, whereas the nuclear localization signal (NLS) is in red. Repressor regions of the protein are represented with R. The basic helix-loop-helix (bHLH) domain and the C domain are highlighted in light green and dark green, respectively.

### 3.1 Dysregulation of TCF4 expression

*TCF4* is a widely expressed gene that produces over 90 independent annotated transcripts due to multiple initiation start sites and alternative pre-mRNA splicing (Fig. 4C). With this wide-ranging transcript profile, it is not surprising that the encoded protein isoforms have been identified in many tissues and historically referred to in the literature by various names (herein referred to as TCF4, but also synonymous with E2-2, ITF2, SEF2, SEF2-1, SEF2-1a, SEF2-1b, PTHS, bHLHb19, MGC149723 and MGC149724) (Corneliussen et al., 1991; Henthorn et al., 1990; Murre et al., 1989; Pscherer et al., 1996; Yoon and Chikaraishi, 1994). At least 18 different N-terminal sequences have been identified and currently 61 unique validated protein-coding isoforms ranging in size from 511 to 773 amino acids are reported in Ensembl (Fig. 4C) (Sepp et al., 2011; Zerbino et al., 2018). Importantly, the protein encoded by *TCF4* should not be confused with the product of *TCF7L2*, transcription factor 7-like 2, which has historically also been referred to as TCF4 in the literature (Hrckulak et al., 2016).

TCF4 is a member of the E-protein transcription factor family that binds to the Ephrussi (E-box) DNA sequence (CANNTG) (Massari and Murre, 2000). Common to nearly all TCF4 isoforms is the presence of a basic helix-loop-helix (bHLH) region (Fig. 4D) (Corneliussen et al., 1991). The bHLH region of TCF4 is highly conserved from mouse (ENSMUSG00000053477) to humans (ENSG00000196628) and serves as an interface with not only DNA, but also with other proteins (Ellenberger et al., 1994; Longo et al., 2008). This region, along with a conserved C domain on the C-terminal end of the bHLH domain (Goldfarb et al., 1998), enables homo- and heterodimerization with other transcription factors or transcription modifiers. TCF4 can potentially dimerize with any of its own isoforms and with any member of the class II, V, and VI bHLH family of transcription factors (Forrest et al., 2014), enabling transcriptional regulation of a number of genes.

In addition to the bHLH region, TCF4 also contains several additional protein domains, depending on the isoform. The presence of transactivation domains (AD1 and AD2) enables binding of additional regulatory molecules that can further modify functions of TCF4 isoforms (Fig. 4). For example, TCF4 interacts with transcriptional activator p300 (*EP300*) via the AD1 domain (Bayly et al., 2004; Massari et al., 1999). However due to alternative splicing, not all TCF4 isoforms contain AD1. Additionally, the presence or absence of nuclear localization signals, export signals and repressor regions can all direct and modify the activity of specific TCF4 isoforms within the cell (Greb-Markiewicz et al., 2019). Of the numerous TCF4 isoforms identified, isoforms A and B are the most extensively characterised (Fig. 4C) (Corneliussen et al., 1991; Skerjanc et al., 1996; Sobrado et al., 2009). While the TCF4-B isoform contains the full complement of activation domains, nuclear localization and export signals, the TCF4-A isoform lacks AD1 and the nuclear localization signal found in the N-terminus (Fig. 4D). However, both still function within the nucleus with TCF4-A relying on heterodimerization with cytoplasmic proteins to cross the nuclear membrane (Sepp et al., 2011). The numerous isoforms and functional regulators contained within the gene underlie the diverse functions of TCF4 in human development and disease. This plasticity illustrates the possibility that dysregulation of a distinct subset of isoforms could contribute to the pathology of FECD within the corneal endothelium.

Genetic variations located within non-coding regions of genes have the potential to function as *cis*-regulators, affecting expression of nearby transcripts by either suppressing or enhancing transcription (Bidichandani et al., 1998; Liu et al., 2012; Todd et al., 2010). This phenomenon has been well documented to contribute to the pathology of several non-coding repeat expansion diseases (Botta et al., 2007; Haeusler et al., 2014; O'Hearn et al., 2015). Likewise, expansion of CTG18.1 has the potential to influence the expression of many of the TCF4 isoforms characterised to date (Fig. 4C). Importantly however, complete haploinsufficiency of TCF4 is known to result in Pitt-Hopkins syndrome (OMIM # 610954); a severe neurodevelopmental disorder associated with profound intellectual disability, absence of speech, behavioural issues and ventilation abnormalities (Amiel et al., 2007; Brockschmidt et al., 2007). It therefore is highly unlikely that expansion of CTG18.1 induces TCF4 haploinsufficiency, given the completely different phenotypes associated with FECD and Pitt-Hopkins Syndrome. However, it is possible that CTG18.1 expansion induces up or down dysregulation of a subset of *TCF4* transcripts. Notably, for approximately half the annotated protein coding *TCF4* transcripts (34/64), CTG18.1 is located within an intron. Additionally, for a few of the isoforms, the CTG18.1 repeat sequence is located within putative promotor regions. The remaining transcripts do not encompass CTG18.1 (Fig. 4). Consequently, it seems logical that if expansion of CTG18.1 does induce dysregulation, only certain transcripts will likely be affected and the effects may be disparate for different isoforms depending on the location of the repeat element within each respective transcript.

Several approaches have been employed to determine if CTG18.1 expansion alters *TCF4* expression within the corneal endothelium (Foja et al., 2017; Mootha et al., 2015; Okumura et al., 2019b; Oldak et al., 2015; Wieben et al., 2018). Oldak et al. used a quantitative (q)-PCR-based approach to explore *TCF4* expression levels between corneal endothelial cell monolayers derived from control subjects and FECD patients with the rs613872 risk allele. In this study, q-PCR probes used to amplify a region of *TCF4* common to all annotated protein-coding transcripts found no significant differences within the stratified FECD patient samples (rs613872 risk allele) or collectively between FECD patient tissues and controls (Oldak et al., 2015). Similarly, Mootha et al. employed a q-PCR based approach to investigate *TCF4* expression levels between FECD expansion-positive corneal endothelial tissues and controls using primers specific to the constitutively expressed exon encoding the bHLH domain present in all TCF4 protein isoforms (Mootha et al., 2015). Like the Oldak study, they observed no significant differences in expression levels. Conversely, Foja et al. using a single TaqMan probe complementary to an exon spanning region in close proximity to the CTG18.1 region found reduced levels of *TCF4* expression within corneal endothelial explants derived from expansion-positive (≥50 repeats) FECD patients when compared to controls (<50 repeats) (Foja et al., 2017). More recently, Okumura et al*.* applied a q-PCR based approach using a combination of primers spanning the canonical *TCF4* transcript (ENST00000354452.8) and found *TCF4* transcription was significantly upregulated in CTG18.1 expansion-positive corneal endothelium (Okumura et al., 2019b).

Given the large number of *TCF4* transcripts characterized to date (Fig. 4), it is perhaps not surprising that studies comparing *TCF4* transcripts from FECD and control corneal endothelial cells have yielded inconclusive results. Opposing results are likely attributed, in part, to the varying specificity of probes used to detect *TCF4* expression by the different studies. Because CTG18.1 expansions likely influence only a subset of total transcripts, experiments designed to quantify total *TCF4* transcripts may be ineffective at detecting subtle isoform-specific dysregulation events. Comprehensive knowledge of all *TCF4* transcripts expressed within the corneal endothelium, and their relative abundance within control and CTG18.1 expansion-positive patients, needs to be determined in order to understand the effect of CTG18.1 expansions on *TCF4* expression. It is anticipated that RNA-Seq based approaches will facilitate this goal by enabling researchers to view *TCF4*-specific transcriptome signatures irrespective of probe bias. Given the diverse array of *TCF4* isoforms, we anticipate third generation long-read transcriptome sequencing will prove most effective by providing end-to-end sequencing of full-length transcripts. This will overcome the reliance on algorithms to piece together short-read data to infer isoform abundance, which is notoriously difficult to accurately achieve for genes such as *TCF4* that comprise so many different transcripts (Fig. 4) (Hardwick et al., 2019).

The importance of determining the effect of CTG18.1 expansion on TCF4 expression is magnified by the fact that several convergent lines of evidence suggest that TCF4 is a regulator of epithelial mesenchymal transition (EMT), a complex process involving epithelial cells depolarizing and adopting motile and invasive properties (Forrest et al., 2013; Sobrado et al., 2009). The process of endothelial mesenchymal transformation (EndMT), as seen in the corneal endothelium, is thought to be regulated by similar pathways and corneal endothelial cells commonly undergo EndMT when cultured in vitro (Roy et al., 2015). Another distinctive inherited corneal endothelial disease, posterior polymorphous corneal dystrophy, is characterized by corneal endothelial cells adopting aberrant epithelial-like features. We, and others, have established that posterior polymorphous corneal dystrophy is associated with dysregulation of EMT governing transcription factors *ZEB1*, *OVOL2* and *GRHL2* (Davidson et al., 2016; Krafchak et al., 2005; Liskova et al., 2018). In light of this and the fact that TCF4 is an established EMT regulator (Sobrado et al., 2009), it is tempting to speculate that dysregulation of TCF4's role within this pathway may contribute, in part, to the pathophysiology of CTG18.1 expansion-mediated FECD. Future adaptation of cell and animal-based model systems will be necessary to comprehensively address this hypothesis.

### 3.2 RNA-mediated toxicity

Repeat-associated RNA toxicity mechanisms are a key driver of pathology underlying DM1 (myotonic dystrophy, type 1; OMIM # 160900) and DM2 (myotonic dystrophy, type 2; OMIM # 602668) (Sznajder and Swanson, 2019), neurological diseases attributed to trinucleotide repeat expansions in non-coding regions of *DMPK* and *CNBP* respectively. Stable RNAs derived from each of the repeat expansions (CUG for DM1 and CCUG for DM2) have been shown to adversely affect developmentally regulated splicing factors in a tissue-specific manner leading to neuromuscular dysfunction (Braz et al., 2018; Matloka et al., 2018; Mohan et al., 2014). Toxicity arises when transcribed RNA containing repeat sequences accumulate in the nucleus of affected cells and sequester RNA binding proteins. These RNA binding proteins, including splicing factor muscleblind-like (MBNL) proteins, are sequestered in ribonucleoprotein structures known as RNA foci [reviewed in (Sznajder and Swanson, 2019)]. Sequestration of MBNL proteins results in an imbalance of available MBNL proteins and CUG binding protein 1 (CUGBP1), causing wide-spread changes in pre-mRNA splicing regulation (Kuyumcu-Martinez et al., 2007; Lin et al., 2006). Additional gain-of-function RNA toxicity mechanisms have been proposed including miRNA processing (Perbellini et al., 2011), transcriptional dysregulation (Botta et al., 2007) and global translational inhibition through stress granule induction (Huichalaf et al., 2010; Onishi et al., 2008). Evidence from in vitro cell experiments and studies performed in a mouse model of DM1 also indicate that MBNL depletion can influence mRNA localization and stability (Sobczak et al., 2013; Wang et al., 2012).

**Fig. 5:**
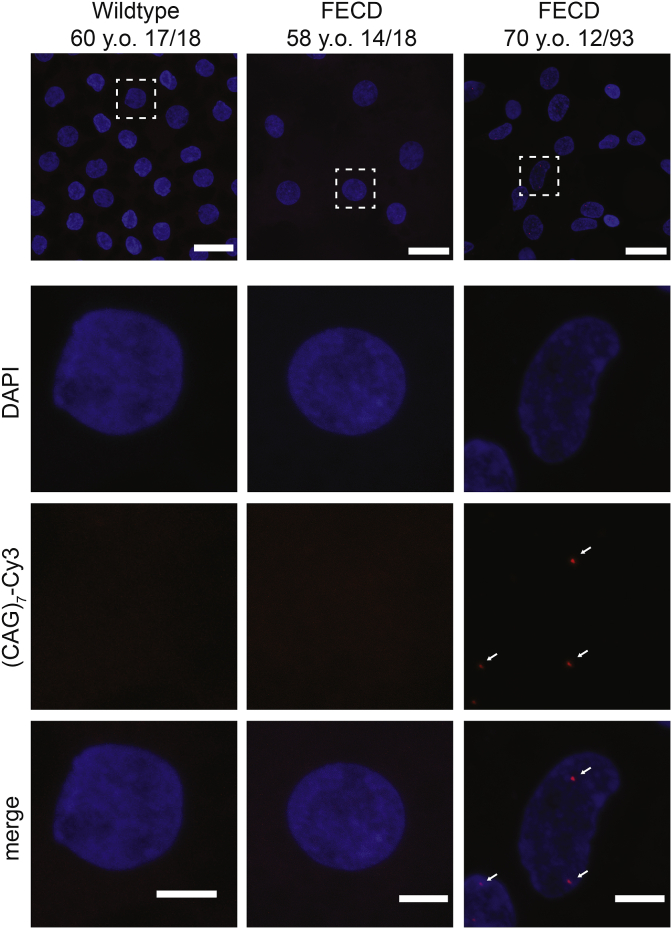
**Representative fluorescence *in situ* hybridization (FISH) images of healthy control, CTG18.1 expansion-negative Fuchs endothelial corneal dystrophy (FECD) and CTG18.1 expansion-positive FECD corneal explant tissue.** (CUG)_n_ RNA foci detection was performed using Cy3-(CAG)_7_ probe using methods adapted from Zarouchlioti et al., (2018). White arrows highlight RNA foci within the CTG18.1 expansion-positive endothelium. y.o., years old. Scale bar for multiple nuclei, 20 μm (top panel). Single nuclei, 5 μm (lower panels).

**Fig. 6:**
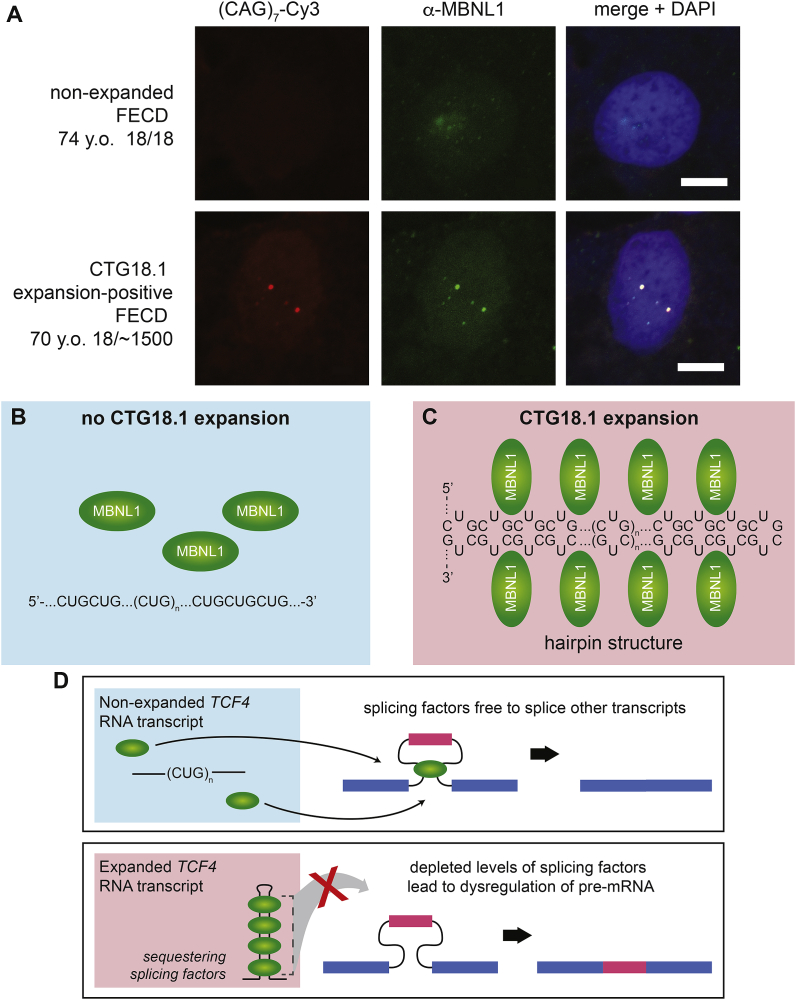
**Sequestration of MBNL1 to RNA foci within corneal explant tissue.** A. MBNL1 is sequestered to the RNA foci within the corneal endothelium of only CTG18.1 expansion-positive FECD patient-derived tissues, scale bar 5 μM. B. Schematic illustrating that in the absence of CTG18.1 expansions, MBNL1 is soluble within the nucleus. C. In the presence of a CTG18.1 expansion, levels of functional MBNL1 are depleted due to the sequestration of the splicing factor to hairpin structures formed by the RNAs comprising expanded copies of *TCF4* transcripts. D. Schematic depicting hypothesised mechanism of splicing dysregulation observed within expansion-positive corneal endothelial cells where the sequestering of splicing factors to hairpin structures formed by *TCF4* RNA transcripts results in widespread dysregulation of pre-mRNA splicing. Permission for re-use of (A) from Du et al. (2015) was granted through CC-BY license (https://creativecommons.org/licenses/by/4.0/legalcode).

The insights gained from the well-characterised RNA-mediated toxicity mechanisms associated with DM1 and DM2 have driven understanding of similar gain-of-function effects mediated by CTG18.1 expansions in FECD. We and others have identified the presence of stable sense-derived (CUG)_n_ and antisense-derived (CAG)_n_ RNA transcripts in FECD patient-derived corneal endothelial cells (Fig. 5) (Du et al., 2015; Hu et al., 2018, 2019; Rong et al., 2019; Zarouchlioti et al., 2018). We have found that these RNA aggregates act as a consistent biomarker for CTG18.1 expansion-mediated FECD and are highly concordant with genotype status (Zarouchlioti et al., 2018). Furthermore, we demonstrated that sense-derived (CUG)_n_ foci consistently sequester MBNL proteins and correlate with CTG18.1 expansion status (Fig. 6) (Du et al., 2015; Zarouchlioti et al., 2018). However, antisense-derived (CAG)_n_ foci appear to be less abundant than the sense counterparts in corneal endothelial explants (Hu et al., 2018) and evade detection in primary corneal endothelial cell cultures (Zarouchlioti et al., 2018).

Evaluation of pre-mRNA splicing patterns using MISO (Mixture of Isoforms) followed by PCR validation in corneal endothelial tissues obtained from two limited datasets of FECD patients and controls enabled us to identify 24 events that were strongly concordant with CTG18.1 expansion status and FECD (Table 4) (Wieben et al., 2017). We found that dysregulated pre-mRNA splicing of *NUMA1*, *PPFIBP1*, and *MBNL1* represented the most robust and consistent signatures of dysregulation associated with CTG18.1 expansions. Intriguingly many of these aberrantly regulated splicing events are common to those defined previously in DM1 (Nakamori et al., 2013; Wang et al., 2012).

**Table 4:** **Dysregulated pre-mRNA splicing events associated with CTG18.1 expansion-mediated Fuchs endothelial corneal dystrophy (FECD).** RNA-Seq data generated from corneal endothelial tissues isolated from FECD patients and controls was analysed with MISO (Mixture of Isoforms) to identify dysregulated pre-mRNA splicing events. PCR was used to valid MISO-identified events in independent corneal endothelial tissues from FECD patients and controls. In total, 24 events were strongly concordant with CTG18.1 expansion status in Fuchs endothelial corneal dystrophy.

Gene	Genomic location of alternative pre-mRNA splicing event (GRCh37)		
	Upstream (5′) exon	Differentially-spliced exon	Downstream (3′) exon
*ABI1*	chr10:27065994:27066170	chr10:27060004:27060018	chr10:27059174:27059274
*ADD3*	chr10:111890121:111890244	chr10:111892063:111892158	chr10:111893084:111895323
*AKAP13*^a^	chr15:86198648:86199018	chr15:86201768:86201821	chr15:86207794:86207986
*TSPOAP1*	chr17:56387328:56387519	chr17:56385902:56386741	chr17:56385203:56385302
*CD46*	chr1:207958964:207959027	chr1:207963598:207963690	chr1:207966864:207968861
*CLASP1*	chr2:122204913:122205083	chr2:122203025:122203072	chr2:122187649:122187753
*COPZ2*	chr17:46105838:46105876	chr17:46105042:46105155	chr17:46103533:46103841
*EXOC1*	chr4:56749989:56750094	chr4:56755054:56755098	chr4:56756389:56756552
*FGFR1*	chr8:38314874:38315052	chr8:38287200:38287466	chr8:38285864:38285953
*GOLGA2*	chr9:131036129:131036251	chr9:131035064:131035144	chr9:131030699:131030803
*INF2*	chr14:105180540:105181193	chr14:105181621:105181677	chr14:105185132:105185947
*ITGA6*	chr2:173362703:173362828	chr2:173366500:173366629	chr2:173368819:173371181
*KIF13A*	chr6:17794480:17794626	chr6:17790103:17790141	chr6:17788007:17788106
*KIF13A*^a^	chr6:17772139:17772290	chr6:17771345:17771449	chr6:17763924:17765177
*MBNL1*^a^^b^	chr3:152163071:152163328	chr3:152164493:152164546	chr3:152165409:152165562
*MBNL2*^b^	chr13:97999058:97999321	chr13:98009050:98009103	chr13:98009736:98009889
*MYO6*	chr6:76618213:76618344	chr6:76621389:76621415	chr6:76623780:76623998
*NHSL1*	chr6:138768138:138768330	chr6:138763120:138763251	chr6:138751530:138754817
*NUMA1*^b^	chr11:71723941:71727306	chr11:71723447:71723488	chr11:71721832:71721900
*PLEKHM2*	chr1:16046229:16046415	chr1:16047824:16047883	chr1:16051812:16052040
*PPFIBP1*	chr12:27829361:27829532	chr12:27829997:27830029	chr12:27832422:27832572
*SCARB1*	chr12:125270903:125271049	chr12:125267229:125267357	chr12:125262174:125263132
*SYNE1*	chr6:152469180:152469513	chr6:152466622:152466690	chr6:152464758:152464900
*VEGFA*	chr6:43746626:43746655	chr6:43749693:43749824	chr6:43752278:43754223

a Aberrant splicing event identified to be specific to CTG18.1 expansion status in the presence of FECD phenotype only (Wieben et al., 2019).

b aberrant splicing event independently validated by RT-PCR in primary corneal endothelial cells derived from FECD patients (Zarouchlioti et al., 2018); modified from and used with permission from Wieben et al. (2017).

To further investigate the biological relevance of aberrant pre-mRNA splicing events associated with FECD pathogenesis, we also investigated a small cohort of elderly patients with CTG18.1 expansions (ranging from 67 to 83 repeats) but without an FECD phenotype (Wieben et al., 2018). RNA-Seq analysis of transcripts isolated from the corneal endothelium of these patients revealed that the majority of pre-determined CTG18.1 expansion-associated pre-mRNA splicing changes persisted in these individuals, despite the absence of FECD disease. However, pre-mRNA splicing events affecting *MBNL1*, *KIF13A* and *AKAP13* were all identified to be unique to the FECD phenotype-positive group suggesting that these events may be drivers of FECD pathology. The identification of normally spliced MBNL1 present in individuals with CTG18.1 expansion in the absence of FECD is of particular interest. A recent study suggested that lower levels of MBNL1 are found in human corneal endothelial cells compared to other human cell types, making depletion of MBNL1 via sequestration a viable explanation for the tissue specificity of the disease (Rong et al., 2019). It is thus reasonable to speculate that having sufficient levels of MBNL1 to perform crucial pre-mRNA splicing may be an essential event that effectively helps maintain normal corneal endothelial cell status. However, additional studies will be required to examine this interesting observation more closely.

In addition to dysregulated pre-mRNA splicing of various genes, we have established that expansion of CTG18.1 within the corneal endothelium results in retention of the *TCF4* intron encompassing the repeat. The direct relationship between *TCF4* intron-retaining transcripts and the presence of RNA foci is yet to be understood (Du et al., 2015; Sznajder and Swanson, 2019; Sznajder et al., 2018; Wieben et al., 2019a). It is possible that currently unidentified mis-spliced *TCF4* transcripts could lead to translation of unique and potentially pathogenic TCF4 isoforms. In Huntington's disease, the mis-splicing of intron 1 in *HTT* transcripts results in a short polyadenylated mRNA corresponding to a short exon 1 protein product that is highly pathogenic (Gipson et al., 2013). Future approaches exploring CTG18.1 expansion-specific *TCF4* transcriptome signatures of dysregulation will hopefully address if similar mechanisms are involved in FECD pathogenesis.

Intron retention acts as a biomarker of CTG18.1 expansion in corneal endothelial cells (Du et al., 2015; Sznajder et al., 2018). However, we have demonstrated that this mechanism is not sufficient to cause disease given that RNA derived from corneal endothelial cells of elderly individuals with CTG18.1 repeat expansion in the absence of FECD phenotype had comparable levels of intron retention to affected FECD patients (Wieben et al., 2019b). Intron retention is more broadly recognized as a feature of CG-rich intronic expansions, as is the case for *C9orf72*-associated ALS (Niblock et al., 2016) and CCHC-Type Zinc Finger Nucleic Acid Binding Protein (CNBP)-associated DM2 (Raheem et al., 2010; Sznajder et al., 2018). Such events are detectable in both affected corneal endothelial tissue and peripheral blood lymphocytes, serving as a biomarker for the detection of individuals with CTG18.1 expansion (Du et al., 2015; Sznajder et al., 2018; Wieben et al., 2019b).

Our data suggests that RNA toxicity induced by CTG18.1 expansions is a compelling mechanism to explain FECD pathology, given the strong concordance between RNA foci, MBNL sequestration and the resulting dysregulated splicing events consistently observed in expansion-positive patients (Du et al., 2015; Zarouchlioti et al., 2018). However, there remains much to be learned about this pathomolecular mechanism and its contribution to FECD pathology. Currently, we do not understand the tissue specificity of this process, and the presence of sense-derived (CUG)_n_ foci has only been investigated in patient-derived CTG18.1 expansion-positive fibroblasts and corneal endothelial cells. While the existence of RNA foci in corneal endothelial cells has been confirmed by several groups, similar studies have seen contrasting results in fibroblasts. We have identified foci in some CTG18.1 expansion-positive fibroblasts, but not in others (Du et al., 2015; Zarouchlioti et al., 2018). These differences may be attributed to cell-type specific thresholds with respect to repeat size and foci occurrence. However, it is not clear why the corneal endothelium is so susceptible to disease, despite *TCF4* being broadly expressed across diverse cell types. Similarly, we do not understand if recruitment of MBNL and other RNA binding proteins solely impacts pre-mRNA splicing regulation. In DM1, sequestration of MBNL proteins also interferes with mRNA localization, stability and other diverse RNA toxicity-associated mechanisms (Wang et al., 2012). Future examination of the impact of RNA foci development, MBNL sequestration, the impact of dysregulated splicing and more broadly differential gene expression at the molecular and physiological levels will be necessary to understand their importance in disease development. This understanding will aid in the development of therapeutic targets to prevent and/or slow disease progression.

### 3.3 Repeat-associated non-AUG dependent (RAN) translation

**Fig. 7:**
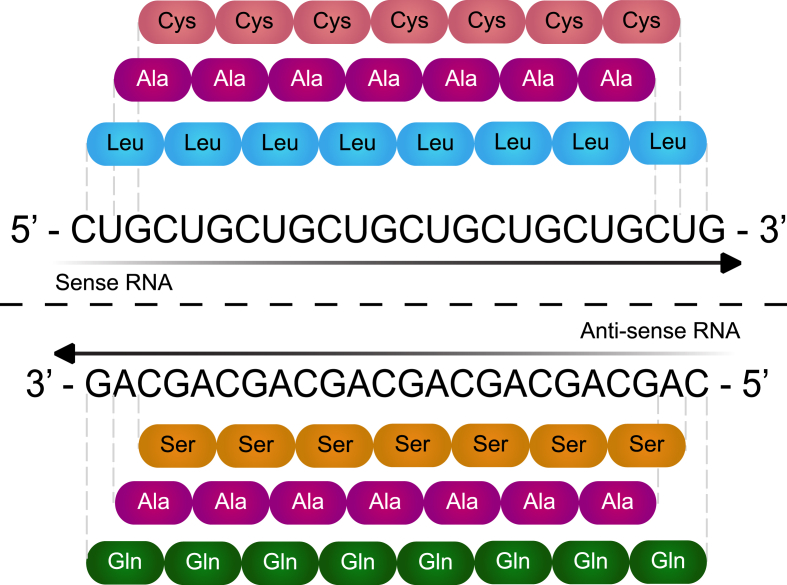
**Poly-peptide proteins potentially translated from CTG18.1 expansions.** Illustration of the potential repeat peptides that may be generated by repeat-associated non-AUG (RAN) translation from expanded (CUG)_n_ RNA transcripts. The sense strand sequence may generate poly-Leucine (Leu), poly-Alanine (Ala) and poly-Cysteine (Cys) peptides and the antisense strand sequence may generate poly-Glutamine (Gln), poly-Alanine (Ala) and poly-Serine (Ser) peptides.

In 2011, Zu et al. made a ground-breaking discovery that homopolymeric proteins could be synthesized from repetitive RNA transcripts in the absence of an AUG start codon; a process called RAN translation (Zu et al., 2011). RAN translation has been implicated in several repeat-mediated diseases including spinocerebellar ataxia 8 (SCA8) (Zu et al., 2011), *C9ORF72* ALS/FTD (Ash et al., 2013; Mori et al., 2013; Zu et al., 2013), DM1 (Zu et al., 2017) and DM2 (Zu et al., 2017). As explained above, corneal endothelial cells derived from FECD patients containing a CTG18.1 expansion transcribe both stable sense-derived (CUG)_n_ and antisense-derived (CAG)_n_ RNA transcripts (Du et al., 2015; Hu et al., 2018, 2019; Rong et al., 2019; Zarouchlioti et al., 2018). Translation of these repetitive CUG and CAG containing RNA transcripts from the *TCF4* gene could theoretically produce five distinct repeat peptides when considering all possible open reading frames (Fig. 7). In other CTG repeat-associated disorders, RAN translation-derived peptides have been shown to form nuclear and cytoplasmic inclusions resulting in varying levels of cell toxicity. Thus, it is conceivable that the presence of RAN translated products originating from the CTG18.1 expansion sequence may contribute to, or drive, the accelerated levels of corneal endothelial cell death observed in FECD (Ayhan et al., 2018; Banez-Coronel et al., 2015; Zu et al., 2011).

With the aim of determining whether RAN translated peptides are produced from the CTG18.1 expansion containing transcripts, our collaborators generated a series of polyclonal antibodies to identify the putative C-terminal regions of four of the five possible RAN translation derived peptides: poly-Alanine (Ala), poly-Cysteine (Cys), poly-Glutamine (Gln) and poly-Serine (Ser) (Soragni et al., 2018). Use of these antibodies in cells containing transient overexpression of constructs harbouring CTG repeats showed that the CTG18.1 expansion within it's genomic context is transcribed and translated via a non-AUG dependent mechanism. These data support the hypothesis that RAN translation may occur in CTG18.1 expansion-positive tissues. Additionally, these investigators used an anti-peptide antibody raised against the putative C-terminal region of poly-Cys peptide and detected expression of the poly-Cys species within corneal endothelial tissue derived from a CTG18.1 expansion-positive FECD patient. These findings provided the first evidence that RAN translation may be occurring in FECD corneal endothelium and suggested that this mechanism might contribute to FECD pathophysiology. However, additional efforts are needed to build upon these data to further characterize poly-Cys species within FECD tissue. It will also be necessary to determine if other short peptide species including poly-Ala, poly-Leu, poly-Gln and poly-Ser may be present, before beginning to dissect out the individual contribution of each distinct polypeptide to disease. This will not be a trivial task given the custom antibody requirements and the difficulties in detecting RAN translated peptides in human tissues. However, these studies will likely provide important insights that will greatly enhance understanding of the cellular mechanisms driving CTG18.1 expansion-mediated disease and could provide an opportunity for therapeutic development.

### 3.4 Somatic instability of CTG18.1

Disease-associated repeats can increase in length over an individual's lifetime in both an age-dependant and tissue specific manner, thus contributing to the tissue-specificity and symptom progression of a given disease (Morales et al., 2012). The somatic instability of disease-associated repeats therefore serves as an attractive hypothesis to explain the tissue-specificity and phenotypic variability of various repeat-associated diseases including DM1, DM2 and Huntington's disease (Cumming et al., 2018; Long et al., 2017; Monckton et al., 1995; Morales et al., 2012; Ohya et al., 1995; Trang et al., 2015). For example, DM1 patient-derived leukocytes typically have expansions of less than 250 CTG repeats in the *DMPK* gene while affected muscle cells can contain thousands of repeats within the same individual (Anvret et al., 1993). *DMPK* repeat length in muscle tissue has been correlated with age-of-onset and disease severity, with individuals having longer repeat lengths in muscle showing more debilitating symptoms (De Antonio et al., 2016; Dogan et al., 2016; Harper et al., 1992; Jansen et al., 1994; Kinoshita et al., 1996). DNA repair-dependant mechanisms and transcription-coupled nucleotide excision repair mechanisms are hypothesised to underlie this process, given that symptoms of DM1 predominantly occur in post-mitotic tissues (heart, skeletal muscle and the central nervous system) (Foiry et al., 2006; Morales et al., 2016; Seriola et al., 2011; Slean et al., 2008; Wohrle et al., 1995). In Huntington's disease, age-related oxidative stress has similarly been shown to lead to tissue-specific increases in expanded repeats via impaired DNA repair mechanisms (Kovtun et al., 2007).

Utilizing a long-read amplification free sequencing method (discussed in section 2.5), we demonstrated that expanded CTG18.1 alleles display high levels of somatic instability (Hafford-Tear et al., 2019). Analysing genomic DNA derived from CTG18.1 expansion-positive FECD patient leukocytes, expanded CTG18.1 repeats were found to behave dynamically with single DNA molecules harbouring both expanded and contracted copies of CTG18.1 repeat length. Notably, greater levels of instability were found to be positively correlated with increased CTG18.1 length. However, we have yet to establish how CTG18.1 behaves within the affected corneal endothelium. Despite the lack of understanding, it is tempting to speculate that expanded CTG18.1 alleles within corneal endothelial tissue will be prone to higher levels of somatic expansion and thus drive FECD progression, especially considering corneal endothelium's post-mitotic state and lifelong exposure to ultraviolet light. If DNA mismatch repair-dependant mechanisms are identified to be causal, it will be important for future research to probe how regulation of this process correlates with phenotypic outcomes (Ciosi et al., 2019; Consortium, 2015). It also remains plausible in some instances that somatic instability of CTG18.1 could be attributed to DNA replication errors occurring during early embryogenesis (McMurray, 2010).

## 4 Model systems and tools for studying CTG18.1 expansion-mediated FECD

Complementary uses of patient-derived tissue and in vitro cell-based systems have so far been employed to enhance understanding of CTG18.1 expansion pathology. However, there is currently a complete lack of animal models to probe CTG18.1 expansion-mediated disease mechanisms. In this section we highlight progress made, limitations facing different model systems, and the need to develop animal models to enhance our understanding of FECD disease mechanisms for rapid translational advances.

### 4.1 Histopathology

Examination of histopathological specimens in combination with in vivo imaging techniques have historically been important methods that informed the field regarding the structural and morphological changes that occur in FECD. Unlike other repeat-expansion diseases, FECD is a common disease affecting an accessible tissue that can be visually or microscopically monitored for pathological and sub-symptomatic changes. Additionally, the diseased tissue is often surgically removed in advanced cases as part of standard patient care, making tissue available for examination. The availability and accessibility of diseased patient tissue offers researchers an opportunity to study endogenous mechanisms within relevant genomic and cellular context. However, these approaches are limited by the fact that tissue is generally excised in patients with end-stage disease and does not capture early pathogenic events.

Despite histopathological changes being well characterized for FECD, such findings are yet to be effectively correlated with genotypes. Studies investigating whether histological differences exist between CTG18.1 expansion-positive and CTG18.1 expansion-negative FECD cohorts, or whether correlations exist between CTG18.1 expansion length and zygosity status have not been performed. As our understanding of pathogenic mechanisms develops, it is anticipated that surgically explanted tissue will be valuable to understand and validate disease mechanisms. It is also anticipated that investigating biomarker distributions (e.g. foci occurrence) across varying cell types within and beyond the eye may help explain the tissue specificity of the disease.

### 4.2 Human corneal endothelial cell lines

**Fig. 8:**
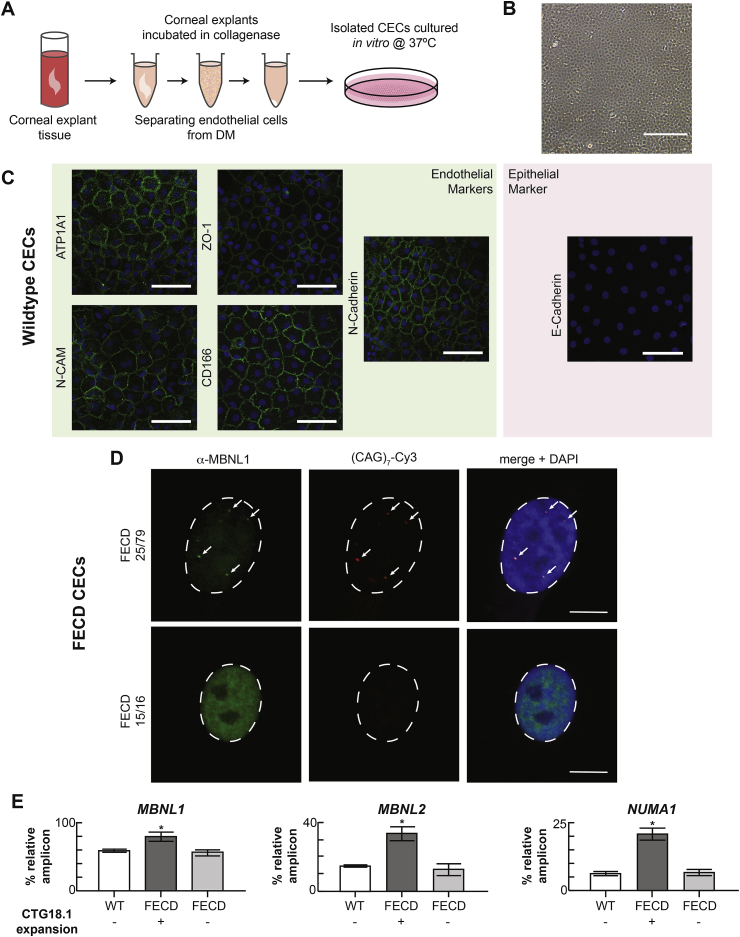
**Primary corneal endothelial cell (CEC) cultures provide an in vitro system to model CTG18.1 expansion-associated Fuchs endothelial corneal dystrophy (FECD) and display key biomarkers synonymous with CTG18.1-expansion associated FECD within corneal explant tissue.** A. Schematic depicting the approach to culture primary human CECs from corneal explant tissue. B. Phase contrast image of primary CECs in culture displaying hexagonal morphology. Scale bar, 0.4 mm. C. Primary CEC cultures displaying distinctive corneal endothelial polygonal morphology and expressing markers indicative of endothelial cell status. Endothelial markers N-Cadherin, ZO-1, ATP1A1, N-CAM and CD166 were detected and the epithelial marker E-Cadherin was absent in CEC lines derived from healthy explant tissue. Scale bars, 100 μm. D. Representative images of MBNL1 protein nuclear localization in CEC derived from CTG18.1 expansion-positive FECD-affected subjects and CTG18.1 expansion-negative FECD-affected subjects. RNA foci are labelled with a Cy3-(CAG)_7_ probe and DAPI is used to stain nuclei. Scale bars, 10 μm. E. Aberrantly regulated pre-mRNA splicing events are detected within CTG18.1 expansion-positive primary CECs. Graphs presented represent the mean percentage expression of amplicons of interest relative to total amplified products, per reaction, for each respective group for MBNL1, MBNL2, and NUMA1 transcripts. Error bars represent ±1 standard deviation. P values were calculated by one-way analysis of variance (ANOVA); ∗P < 0.001. Permission for re-use of adapted figure from Zarouchlioti et al., (2018) was granted through CC-BY license (https://creativecommons.org/licenses/by/4.0/legalcode).

Numerous studies have utilized patient-derived primary and immortalised corneal endothelial cell lines to study FECD disease mechanisms and test targeted therapies (Fig. 8A and B) (Du et al., 2015; Hatou and Shimmura, 2019; Mootha et al., 2015; Peh et al., 2015; Zarouchlioti et al., 2018). These cell lines have enabled researchers to investigate the downstream cellular consequences of the CTG18.1 expansion within a relevant genomic and cellular context. Patient-derived corneal endothelial cell lines offer a clear advantage when compared to the adaptation of cellular or animal models that rely on overexpression of repeat elements within artificial genomic contexts and cellular re-programming that cannot fully account for the age-related and cell-type dependent aspects of the disease aetiology.

Primary corneal endothelial cell cultures maintain many in vivo properties with respect to their morphology, transcriptome profiles and behaviour (Fig. 8C). Likewise, corneal endothelial cell lines derived from CTG18.1 expansion-mediated FECD have RNA foci and display many of the aberrantly regulated splicing events that have been described in corneal endothelial tissue (Fig. 8D and E). Given that human adult corneal endothelial cells in vivo are largely non-proliferative cells arrested in the G1 phase of the cell cycle, primary culture methods have been developed to enhance their proliferation while still maintaining endogenous properties of the cells (Joyce et al., 2002; Peh et al., 2015). Nonetheless, primary corneal endothelial cell lines have minimal proliferative capacity and in general can only be used for a finite number of passages and experiments, making their general use labour intensive and limited. Furthermore, any experimental approach that requires a large number of cells is not feasible in this system (Peh et al., 2015; Zarouchlioti et al., 2018). Immortalization of corneal endothelial cell cultures overcomes these hurdles but, in the process, loses some of the morphology and transcriptome profiles of innate corneal endothelial cells. On this basis alone, they have limited utility for experimental approaches that require endogenous corneal endothelial cell properties to be maintained. Nevertheless, immortalised cell lines will still likely continue to prove valuable for more high-throughput screening approaches (Hu et al., 2018, 2019).

A global shortage of appropriate donor tissue to treat corneal endothelial cell failure is driving an international effort to develop protocols that enable transplant grade corneal endothelial cell monolayers from induced pluripotent stem (iPS) cells and embryonic stem (ES) cells. To date, a variety of approaches have been published, exhibiting varying levels of success (Chen et al., 2015; McCabe et al., 2015; Wagoner et al., 2018; Zhang et al., 2014). Using the method by McCabe et al., we recently derived corneal endothelial cells from iPS cells with the aim of modelling the transcriptome effects of an FECD disease-associated *SLC4A11* variant (Brejchova et al., 2019). Despite being able to analyse the corneal endothelial cell-specific effects of the *SLC4A11* variant on pre-mRNA splicing, we were only successful in culturing mixed cell populations using this protocol. Currently, development of stem cell derived corneal endothelial-like cells is hindered by the lack of specific corneal endothelial cell markers and the inability to derive and characterize homogenous cell populations (Hatou and Shimmura, 2019). As development of stem cell-derived corneal endothelial-like cells advances, it is anticipated that these cells will offer researchers useful tools to model FECD disease mechanisms and test novel therapies.

### 4.3 CTG18.1 expansion-associated animal models of FECD

The relative and differing contributions that TCF4 expression, RNA toxicity, RAN translation and somatic and age-dependent repeat instability may have on FECD pathophysiology are not fully understood (Fig. 3). Unfortunately, there currently are no validated animal models harbouring CTG18.1 expansion for studying these mechanisms in vivo. Development of animal models will likely be critical for delineating the pathological mechanisms associated with CTG18.1 expansion-mediated FECD. In other repeat-associated human diseases, animal models have proven to be an effective way to dissect the relative contributions and differential involvements of alternative disease mechanisms (Balendra and Isaacs, 2018; DeJesus-Hernandez et al., 2011; Renton et al., 2011). However, until a CTG18.1 expansion-mediated FECD model is developed and characterized, the research community must rely on tissue and cell cultures to examine pathological mechanisms.

For the development of CTG18.1 expansion-mediated FECD animal models, several factors must be taken into consideration. First, because CTG18.1 expansion-mediated FECD is an age-related disease, it is important that appropriate strains are utilized. For example, some mouse strains such as C57BL/6N contain genomic mutations that cause retinal degeneration. Utilizing such a mouse strain to develop an FECD model would not be appropriate as the animals develop ocular abnormalities that would complicate interpretation of ocular pathophysiology late in life. Second, despite *TCF4* being highly conserved, the syntenic non-coding region in the mouse genome does not contain a CTG repeat, and therefore ‘humanised’ knock-in models will need to be developed to introduce the repeat sequence. Once this has been established, significant characterization of such a model will be required to verify that it produces expression profiles similar to those found in humans. Recent collaborations between the University of Minnesota and our research team have been successful in the technical aspects of creating such a mouse, but long-term assessment of this potential model will be required to determine if any FECD-associated features develop. Targeted isoform-specific knockdown approaches, overexpression systems dissecting different components of RNA-toxicity, and RAN peptides using cell and animal models, may also provide useful insights into CTG18.1 expansion-mediated FECD disease mechanisms.

## 5 Development of treatments for CTG18.1 expansion-mediated FECD

Corneal transplantation is the primary surgical treatment option for FECD, irrespective of genetic causes or associations. Due to a global shortage of suitable donor tissues, there is a clinical imperative for alternative, and ideally preventative, therapies to be developed for this common and age-related disease. Even with the variation in incidence between FECD and CTG18.1 expansion across ethnic groups, the CTG18.1 expansion is by far the most common genetic variant identified in FECD patients; thus therapies designed to target the pathogenic features of this genetic-subtype will offer benefit to a large number of individuals. For this ambition to be realised, future research efforts need to be invested in improving detection of early disease, in parallel with developing CTG18.1 expansion-targeted therapies. In this section, we briefly summarise available FECD treatments, expand upon the significance and challenges of early disease detection, and lastly highlight emerging trinucleotide repeat-targeted therapies of relevance to CTG18.1 expansion-mediated FECD.

### 5.1 Current treatments for FECD

Corneal transplantation is the preferred treatment option for moderate to advanced FECD disease. Prior to surgery, hypertonic saline (5%) drops or ointment are typically prescribed to draw fluid from the cornea which can reduce the duration of visual blur when applied after wakening (Bourne, 1985; Foulks, 1981; Grayson, 1983). Corneal transplantation removes the thickened and distorted Descemet membrane layer and replaces it with healthy donor tissue with a high endothelial cell density (donor cell density >2000 cells/mm^2^). Until recently, penetrating keratoplasty which involves the removal of all central layers of the cornea was the standard procedure to treat FECD patients. It is a technically successful surgery but has a long visual rehabilitation period, a high probability of significant residual refractive error, and increased susceptibility of the eye to subsequent trauma (Christo et al., 2001; Forstot et al., 1975; Foulks, 1981; Mannis et al., 1997; Meyer and Bobb, 1980; Price et al., 1994; Purcell et al., 1982; Thompson et al., 2003). Currently, endothelial keratoplasty, which selectively replaces only the posterior diseased corneal tissue, is the preferred option because the visual rehabilitation time is significantly shorter than after a penetrating keratoplasty with a predictable residual refractive error (Chen et al., 2008; Patel, 2012; Patel et al., 2009; Price and Price, 2005; Price et al., 2009).

All types of corneal transplantation require access to donor material that is viable and safe to use. This involves consent and retrieval of eye tissue, screening the donor and tissue for possible transmission of microbial contaminants, and appropriate storage prior to distribution. Barriers exist at each stage of the process including reluctance of potential donors to participate in eye donation and the capital expenditure of a central eye bank. It has been estimated that worldwide only one individual in 70 with blinding corneal disease has access to suitable donor material for transplantation (Gain et al., 2016). Given the age-related nature of FECD and the increasing population over 60 years of age, it is likely that the number of individuals who develop visually significant FECD will increase. This anticipated rise in incidence is predicted to further increase global demands for donor tissue. In light of this, international efforts are underway to develop and cultivate in vitro transplant-grade human corneal endothelial cells that can be used to reseed the corneal endothelium and overcome the reliance on donor corneal tissue for transplant surgeries [for review see (Okumura and Koizumi, 2020)]. While the success of this approach is still unknown, it underscores the need for innovative alternative FECD therapies to be developed.

### 5.2 Challenges and opportunities associated with early disease detection

The desirable paradigm for future FECD therapies is for preventative treatments to be administered before significant abnormalities of Descemet membrane and endothelial cell loss have occurred. For this approach to be successful, it will entail screening of the population at risk. In vivo imaging modalities such as retroillumination photography or microscopic imaging of the endothelial layer by specular microscopy or confocal microscopy could be employed to broaden the reach of screening programs (see Fig. 1 for image examples). Additionally, given the strong association of CTG18.1 repeat length and FECD, genetic screening of this repetitive element early in life could be adopted as part of routine healthcare practice. However, the argument for implementing screening of CTG18.1 on a population wide scale will likely only become compelling once effective preventative treatment strategies are developed. A combined screening approach involving image analysis with genetic screening, alongside family history of the disease, will likely provide the most comprehensive way to identify patients with early signs of disease that would most benefit from preventative treatments.

### 5.3 Development of CTG18.1 expansion-directed gene editing

**Fig. 9:**
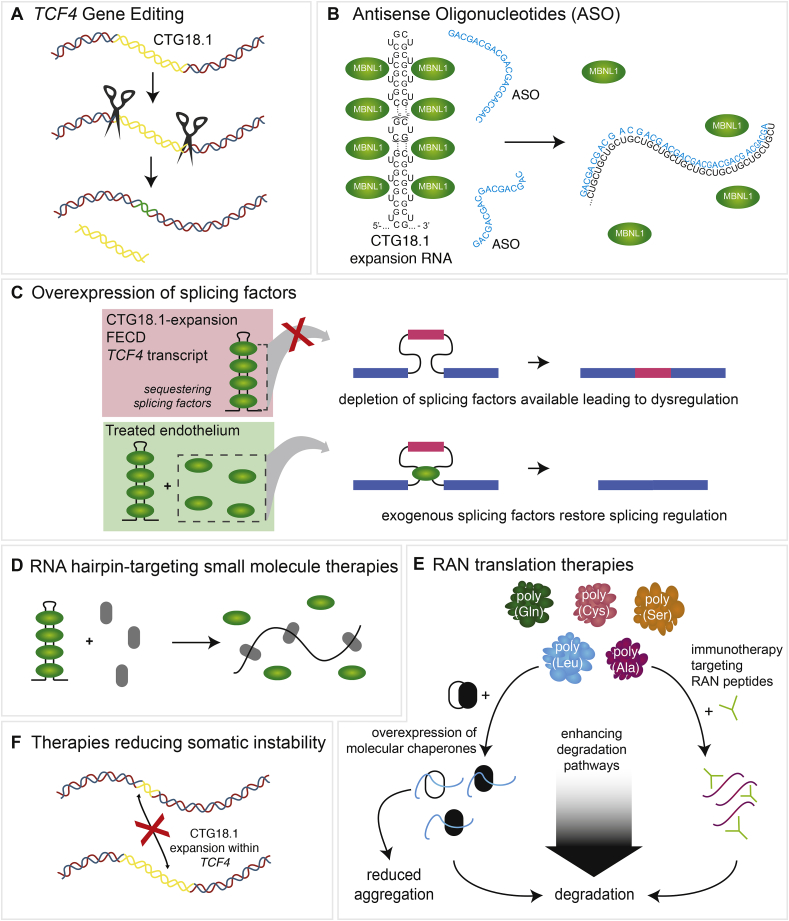
**Potential therapeutic strategies targeting pathogenic mechanisms associated with CTG18.1 expansion-mediated Fuchs endothelial corneal dystrophy (FECD).** A. Gene editing tools may be applied in the future to reduce the size or presence of CTG18.1 expansions. The CTG18.1 repeat element is represented as yellow. B. Antisense oligonucleotide (ASO) therapeutic strategies can target (CUG)_n_ or (CAG)_n_ RNA transcripts derived from expanded copies of CTG18.1 to physically block the formation of foci and or induce degradation of such transcripts. C. Overexpression of splicing factors, such as MBNL proteins, could restore splicing regulation. D. Small molecule therapeutics (grey) could induce disruption of RNA hairpins on (CUG)_n_ or (CAG)_n_ RNA transcripts, releasing sequestered splicing factors (green). E. Various strategies targeting RAN translation may in the future prove to have therapeutic benefit. Immunotherapy targeting RAN peptides would enable the cell to degrade the peptide aggregates. Overexpression of molecular chaperones could potentially prevent the aggregation of RAN peptides and/or enhance degradation levels. F. Therapeutics aimed at reducing levels of somatic and age-related levels of repeat instability may in the future prove to have therapeutic benefit for CTG18.1-expansion associated FECD.

Understanding the pathophysiology of CTG18.1 expansion-mediated FECD will provide the information necessary to develop therapies directed at preventing or delaying pathogenic mechanisms leading to disease. These may include therapies targeted at the genomic level. CRISPR-Cas9 mediated gene editing techniques have become wide-spread over the past few years, and multiple studies have investigated its therapeutic potential for repeat expansion-mediated diseases [see review (Babacic et al., 2019)]. While studies characterizing the effect of genetic editing of the expanded CTG18.1 repeat are yet to be published, the use of targeted deactivated Cas9 to impede repeat transcription in DM1 cells has been shown to diminish the number of repeat containing RNA transcripts in vitro, reducing RNA foci frequency and curtailing aberrantly regulated pre-mRNA splicing events (Pinto et al., 2017). This result is encouraging and conceivably can be tested in vitro in primary or immortalised corneal endothelial cells derived from FECD patients. It is reasonable to speculate that reducing the repeat size may eliminate or reduce the presence of RNA foci, re-establishing normal splicing patterns, and in theory, return the cells to their non-diseased state (Fig. 9A). This approach would be most beneficial in FECD patients with early diagnosis and mild disease. However, it may not be effective as a standalone therapy in moderate and severe disease because it would only treat the remaining corneal endothelial cells. Because loss of corneal endothelial cells is a characteristic of advanced FECD, these patients would likely still require corneal tissue or corneal endothelial cell transplantation to increase numbers so that they can function to appropriately maintain corneal deturgescence.

While gene editing for FECD may appear futuristic at this time, significant progress for its use as a therapeutic for ophthalmic disorders is making its way to the clinic. A phase 1 CRISPR-Cas9 gene therapy clinical trial for Leber Congenital Amaurosis began in 2019 and the first patient has received in vivo therapy to correct a single point mutation within the photoreceptors of the retina (https://www.clinicaltrials.gov/ct2/show/NCT03872479?term=NCT%2303872479&rank=1). As the community waits to hear the success of such an approach, it is important to note that as of publication of this review, no CRISPR-Cas9 based genetic therapies have been approved for use in the clinic. Only one clinical trial performed in the United States has successfully demonstrated an effective use of CRISPR-Cas9 engineered cells, albeit in only 3 cancer patients (Stadtmauer et al., 2020). While this avenue of research and potential therapeutic use appears promising, all gene-editing based approaches need to proceed with caution as lack of specificity of the editing tools used to cleave at precise genomic targets raises questions about their in vivo safety (El-Kenawy et al., 2019).

### 5.4 Development of CTG18.1 expansion-directed therapeutics

In addition to gene editing, modifying agents engineered to disrupt RNA foci formation and MBNL1 sequestration may serve as effective therapeutic strategies for CTG18.1 expansion-mediated FECD (Fig. 9B) (Babacic et al., 2019; Du et al., 2015; Hu et al., 2018, 2019; Pinto et al., 2017; Zarouchlioti et al., 2018). We and others have published several proof-of-concept studies testing the therapeutic potential of antisense oligonucleotides to either block RNA foci from sequestering RNA binding proteins or to degrade them directly (Hu et al., 2018, 2019; Zarouchlioti et al., 2018). These studies have yielded promising effects on downstream features of RNA toxicity within various FECD patient-derived immortalised and primary corneal endothelial cell model systems. However, it is yet to be established if these promising results will be of therapeutic benefit in vivo. It must be kept in mind that patients with a CTG18.1 expansion but no FECD may also have RNA foci in the corneal endothelium, so at least in some patients, altering the number of RNA foci may not be the appropriate target. Future studies testing the efficacy of antisense oligonucleotides aimed at silencing toxic RNA species will be important to understand their potential utility as CTG18.1 expansion-mediated FECD therapeutics for the clinic.

Another target for FECD therapeutic development may be aimed at increasing levels of RNA binding proteins that are sequestered by the CTG18.1 containing RNA foci (Fig. 9C). Given the established link between depleted levels of functional MBNL1 and dysregulated pre-mRNA splicing in CTG18.1 expansion-mediated FECD, it is reasonable to hypothesize that increasing RNA binding proteins such as MBNL1 within the corneal endothelium may have therapeutic benefit. Such an approach has proved successful in reversing RNA mis-splicing and myotonia in mouse models of DM1 (Chamberlain and Ranum, 2012; Kanadia et al., 2006). However, this result is not ubiquitous amongst DM1 mouse models as MBNL1 overexpression has also been reported not to improve skeletal muscle function, cardiac conduction or myotonia within the model, despite rescuing some dysregulated pre-mRNA splicing events linked with depletion of MBNL1 (Yadava et al., 2019).

In addition to the opportunities listed above, several studies have identified small molecules which target and bind RNA hairpins created by repeat expansions (Fig. 9D). Doxorubicin, a nucleic acid intercalating drug was recently shown to effectively disrupt RNA foci in DM1 patient-derived cells (Jain and Vale, 2017). Other agents, such as pentamidine (Chakraborty et al., 2015), furamidine (Jenquin et al., 2018, 2019) and engineered small molecules (Angelbello et al., 2019, 2020; Li et al., 2018; Luu et al., 2016; Reddy et al., 2019; Rzuczek et al., 2017) have shown significant improvement in pathogenic features of CTG repeat expansion-mediated disease in cell and animal models of DM1. It will be interesting to see if similar approaches utilizing FECD-derived corneal endothelial cells and animal models of CTG18.1 expansion-mediated FECD prove to be of therapeutic benefit.

### 5.5 Other FECD therapeutic opportunities

The evidence supporting a role for RAN translation and the potential for these small peptides to induce cellular toxicity in FECD is another area for therapeutic development (Fig. 9E). Studies investigating potential therapies to ameliorate the negative effects of the resultant RAN peptides have been conducted in other repeat expansion-associated diseases such as *C9ORF72* ALS/FTD and SCA8 (Cristofani et al., 2018; Nguyen et al., 2020). These studies involve immunotherapy directed at the RAN peptides and overexpression of chaperones such as small heat shock protein B8 which mediates autophagy of aggregated proteins. These methods have shown positive effects in cells and animal models containing *C9orf72* repeat expansions. Other studies have examined modifying effectors of RAN-mediated nucleocytoplasmic transport to inhibit cytotoxicity due to the aggregation and accumulation of RAN peptides (Boeynaems et al., 2016; Jovicic et al., 2015; Zhang et al., 2015). If future studies confirm RAN translation to be a significant driver of CTG18.1 expansion-mediated FECD pathology, similar therapeutic approaches can be explored.

In a range of repeat-mediated diseases, including Huntington's disease and DM1, polymorphisms within genes encoding DNA mismatch repair regulators have been identified that act as trans-acting genetic modifiers of disease onset and progression to modify somatic instability rates (Ciosi et al., 2019; Morales et al., 2016). Therapeutic manipulation of such polymorphisms or components of DNA mismatch repair pathways are therefore a current area of interest with broad relevance for all repeat mediated disease (Fig. 9F). Notably, a small molecule naphthyridine-azaquinolone has recently been demonstrated to specifically bind the expanded CAG-repeat tracts that cause polyglutamine diseases in Huntington's disease, thereby preventing expansion and encouraging repeat contraction (Nakamori et al., 2020; Nakano et al., 2015). If somatic expansion of CTG18.1 is proven to influence FECD age-of-onset and/or disease progression rates, therapeutic approaches involving repeat structure-specific DNA ligands could also be applicable for FECD.

Regardless of the type of therapy, tissue-specific delivery of the compounds will likely be required to minimise exposure of the given drug to healthy cell types and reduce potential side effects. The localised nature and relative accessibility of the corneal endothelium should enable this goal to be achievable. Non-invasive topical eye drops would likely be the preferred route for drug administration, especially if regular dosing is required. However, this route will require compounds to penetrate the corneal epithelium and stroma to reach the endothelial cells. Furthermore, risks associated with exposure to non-specific cell types or tissues outside of the eye (corneal epithelium, tear ducts, bulbar conjunctiva, inner eyelid) and within the eye (corneal stroma, lens, iris, ciliary body and aqueous outflow pathway) will all need to be addressed.

## 6 Future perspectives

Now that the association between the CTG18.1 expansion and FECD is well established, additional research efforts are required to further probe and understand the key molecular drivers responsible for the disease. Furthermore, future efforts to enhance rates of early disease detection will be paramount to support development of preventative therapies. This section will briefly touch upon some of the outstanding questions that need to be addressed to advance understanding of the mechanisms involved in CTG18.1 expansion-mediated FECD and facilitate the design and development of effective CTG18.1-targeted therapies.

### 6.1 Why is the corneal endothelium specifically vulnerable to CTG18.1 expansion?

The only recognized impact of CTG18.1 expansion on health has been FECD, although altered hearing in undifferentiated FECD patients has been described (Reed et al., 2018; Stehouwer et al., 2011). Other systemic implications of CTG18.1 expansions have not been thoroughly evaluated, so there remains the possibility of clinically important correlations, considering *TCF4* is a widely expressed gene. We do know that expansion of the CTG18.1 repeat sequence impacts the health of the corneal endothelium. However, we do not understand the reason for this tissue-specific effect of CTG18.1 expansions and further research is required to address this important question. As mentioned previously, all CTG18.1 genotyping studies performed to date have used leukocyte-derived DNA to genotype the repeat element; due to technical need for high input genomic DNA which is not feasible in corneal endothelial tissue. It is conceivable that expanded copies of the repeat element within affected corneal endothelial cells are prone to high rates of age-dependent instability, and thus typically contain much larger repeats than patient-matched leukocytes. This has been a characteristic of several repeat expansion disorders. For example, patients with DM1 have repeat expansion sizes in the thousands within *DMPK* in muscle when compared to <250 in blood (Anvret et al., 1993). Additionally, DM1 patients have diverse phenotypes depending on the size of the *DMPK* repeat, with more severe phenotypes correlating to larger repeat expansions in muscle (De Antonio et al., 2016; Dogan et al., 2016; Harper et al., 1992; Jansen et al., 1994; Kinoshita et al., 1996). Current studies also suggest a positive correlation between CTG18.1 repeat size and FECD severity (Eghrari et al., 2017b; Soh et al., 2019, 2020; Soliman et al., 2015), despite these studies only investigating leukocyte specific repeat size. Once the technological advances are in place to interrogate CTG18.1 repeat size within the affected corneal endothelium, it will be important to understand how and if tissue-specific accelerated rates of expansion occur within the corneal endothelium and if so, whether instability rates correlate with disease severity and/or onset.

Another important area of investigation that has only briefly been examined is whether individuals with other repeat expansion diseases are susceptible to FECD. Two independent studies have reported that FECD is a common feature of patients with DM1 in the absence of CTG18.1 expansion suggesting that the presence of a non-coding CTG repeat, regardless of its genomic context (i.e. within *TCF4* or *DMPK*) may be sufficient to cause FECD (Mootha et al., 2017; Winkler et al., 2018). Notably, the presence of FECD in some DM1 families appeared to segregate, further suggesting that FECD is potentially an unrecognised feature of DM1 (Winkler et al., 2018). Additional studies in larger DM1 cohorts are now required to further validate this finding. If confirmed, it would suggest the interesting possibility that expression of expanded CTG repeats, irrespective of genomic context, contribute to the development of FECD via shared pathogenic mechanisms.

### 6.2 Why is CTG18.1-mediated FECD associated with incomplete penetrance and variable expressivity?

Despite the overwhelming evidence for CTG18.1 expansion being a strong risk factor for FECD, we have limited insight regarding the incomplete penetrance and variable expression of the disease. It has been established in different ethnicities that CTG18.1 expansion is present at low levels in control populations (Table 2) and small cohorts of aged individuals in absence of disease (Del-Favero et al., 2002; Foja et al., 2017; Kuot et al., 2017; Luther et al., 2016; Mootha et al., 2014; Nakano et al., 2015; Nanda et al., 2014; Okumura et al., 2019c; Rao et al., 2017; Skorodumova et al., 2018; Vasanth et al., 2015; Wieben et al., 2012; Xing et al., 2014; Zarouchlioti et al., 2018). While some proportion of these control patients may develop FECD later in life, it is clear that some individuals with a CTG18.1 repeat expansion do not. Even within families, penetrance can be incomplete. For example, we identified one family in our cohort where an unaffected father in his 90's was heterozygous with a CTG18.1 expansion allele containing >2500 repeats (in lymphocytes), but had a daughter with a smaller bi-allelic expansion (85 repeats) who developed FECD in her 50's (unpublished data). Whether the presence of CTG18.1 expansion on both alleles is an indicator of earlier disease and disease severity is currently unknown. Only 4.7% and 7.3% of FECD patients recruited at Moorfields Eye Hospital and the Mayo Clinic respectively have been identified with bi-allelic CTG18.1 expansion (unpublished). In our experience, these individuals do not generally present earlier or have more severe disease when compared to subjects harbouring mono-allelic CTG18.1 expansion, however, additional studies are necessary to more comprehensively address this question.

Other mechanisms may also affect FECD penetrance and expressivity. Somatic instability between the affected/non-affected tissues and interruptions within the repeat sequence have both been shown to influence the onset and expressivity of several repeat-mediated diseases (Consortium, 2019; Eichler et al., 1994; Lopez Castel et al., 2011; Mason et al., 2014; Mornet et al., 1996; Santoro et al., 2017). While the need for high genomic DNA input requirements of long-read non-amplification-dependant sequencing technologies currently precludes the measurement of repeat size and instability determination in corneal endothelial cells (discussed in section 2.5), we have used this technique to investigate repeat interruptions and the adjacent CCT repeat motif in leukocyte genomic DNA isolated from individuals (n = 5) harbouring a CTG18.1 repeat expansion but no disease (Wieben et al., 2019a). Our preliminary analysis did not identify any changes in the repeat sequence that would explain why these patients do not get FECD, but it did show the potential value of this technique for addressing this question. Given that our initial investigation was limited, additional studies with greater sample sizes are needed to further examine whether interruptions or polymorphisms encompassed within CTG18.1 expansion sequences influence the expressivity of FECD disease.

### 6.3 Why do women have a predilection for FECD?

Another important aspect of disease variation is the recognized fact that women more commonly express FECD and in general have more severe clinical presentation. This trend is consistent across diverse populations and holds true for groups with and without CTG18.1 repeat expansion (Kuot et al., 2017; Nakano et al., 2015; Nanda et al., 2014; Okumura et al., 2019c; Rao et al., 2017; Skorodumova et al., 2018; Soliman et al., 2015; Vasanth et al., 2015; Wieben et al., 2012; Zarouchlioti et al., 2018). While our current understanding of FECD lends essentially no insight into this observation, several possible explanations exist. These include hormone-related mechanisms, the interplay of other genetic variants either within the *TCF4* gene or other genes, epigenetic factors, environmental influence, and increased life span. For a comprehensive review of FECD female predominance see Ong Tone et al., (2020) (Ong Tone et al., 2020). Understanding the aetiology that underlies the female preponderance for the disease may very well direct the future pursuit of pathogenic mechanisms and therapeutic targets.

### 6.4 What impact does environment have on incomplete penetrance and expressivity of FECD?

In addition to the genetic background of individuals, environmental factors are also likely to influence phenotypic expression of FECD. Smoking has been suggested to double the risk of developing cornea guttae (Zoega et al., 2006) and increase the risk of FECD by 30% (Zhang et al., 2014). Exposure to ultraviolet light has also been suggested as an environmental risk factor for FECD. Recently, exposure of mouse corneas to ultraviolet A light resulted in the development of a non-genetic FECD-like model of the disease. These animals developed corneal edema, loss of corneal endothelial cells and at the molecular level, had an increase in mitochondrial nuclear DNA damage (Liu et al., 2020). Interestingly, female mice had more severe disease than male mice which is similar to human FECD. Further investigation of this model and the impact of other environment factors will be necessary to identify risk factors associated with FECD pathogenesis.

The impact of environmental risk factors on disease has most closely been associated with an increase in oxidative stress. Oxidative stress occurs when the presence of free radicals is in excess to the number of antioxidants, causing a toxic environment that can be harmful to the surrounding cells and tissues. Several lines of evidence support chronic oxidative stress as an underlying mechanism in FECD pathogenesis (Jurkunas, 2018; Ong Tone et al., 2020). Elevated free radicals such as advanced glycation end products, peroxynitrite and lipid peroxidation have been identified in FECD corneas and Descemet's membranes (Buddi et al., 2002; Jurkunas et al., 2010; Wang et al., 2007). Smokers and individuals with increased exposure to ultraviolet light have both been found to have increased levels of reactive oxygen species and decrease antioxidants in aqueous humour and ocular tissues (Cheng et al., 2000; Liu et al., 2016, 2020; Solberg et al., 1998; Zinflou and Rochette, 2017). It is interesting that dysregulation of oxidative stress defences are commonly found in DNA repeat expansion diseases (Browne and Beal, 2006; Cotticelli et al., 2013; Ihara et al., 1995; Maiuri et al., 2019; Ranganathan et al., 2009; Song et al., 2016; Stucki et al., 2016). Furthermore, oxidative stress can also influence repeat expansion length (Chatterjee et al., 2015). If it is found that FECD corneal endothelial cells have a larger CTG18.1 repeat expansion than what is found in leukocytes, the somatic instability within these cells may be attributed in part to environmental influences. Expansion of research efforts in this area is necessary to understand the underlying aetiology in FECD.

### 6.5 What are the key pathogenic underlying mechanisms associated with CTG18.1 expansion-mediated FECD?

A number of molecular mechanisms have been proposed that may be involved in the pathophysiology of CTG18.1 expansion-mediated FECD (Fig. 3). Future insights and mechanistic parallels will continue to be drawn from the non-coding repeat-mediated diseases field. For example, the non-coding hexanucleotide GGGGCC repeat expansion in C9orf72 is recognized to be the most frequent genetic cause of amyotrophic lateral sclerosis (ALS) and frontotemporal dementia (FTD) in Europe and North America (DeJesus-Hernandez et al., 2011; Renton et al., 2011). Three non-mutually exclusive disease mechanisms have been proposed to the underlying condition, similarly to CTG18.1 expansion-mediated FECD: (1) loss of C9orf72 protein function, (2) toxic gain-of-function from repeat-associated RNA transcripts, and/or (3) repeat associated RAN translation of toxic gain-of-function dipeptide repeat proteins (Balendra and Isaacs, 2018). Future research efforts are needed to continue to dissect out the relative contributions of such mechanisms to FECD pathophysiology.

Several cellular biomarkers of CTG18.1 expansion status have now been well characterised including the accumulation of RNA aggregates within affected patient-derived cells, sequestration of MBNL proteins to the foci and a diverse pattern of dysregulated pre-mRNA splicing events. Furthermore, headway has been made to establish the presence of RAN-derived polypeptides within CTG18.1 expansion-positive tissues. While it can be hypothesised how these biomarkers are toxic leading to cellular dysfunction, the relative contribution of the events to FECD development and progression are yet to be established. It is reasonable to postulate that such events may have a cumulative effect and it will take significant future research efforts to dissect their relative contributions.

The impact of CTG18.1 expansion on TCF4 expression in corneal endothelial cells is still unknown. Because TCF4 interacts with many bHLH proteins, it is conceivable that slight changes in the abundance of different isoforms could have significant impact on cell function. Before this can be assessed however, it will be important to identify which TCF4 transcripts are expressed in healthy corneal endothelium and how the distribution of different isoforms is affected by CTG18.1 expansion. This is not a trivial task as more than 90 different TCF4 transcripts have been identified. Future transcriptomic analysis of healthy and disease tissue will be essential to address this important question.

Identification of dysregulated transcriptomic and proteomic features specific to CTG18.1 expansion-mediated FECD should provide valuable insights into the disease. At least 25% of FECD individuals do not have expanded copies of CTG18.1 and the majority of these cases currently lack a molecular diagnosis. As for most genetically heterogeneous diseases, it is likely that specific genetic subtypes will harbour unique mechanism of dysregulation in addition to sharing converging common downstream features, given that all FECD cases with or without a CTG18.1 expansion experience the same phenotypic endpoint: presence of guttae and loss of corneal endothelial cells. We anticipate that this subgroup of individuals (i.e. FECD patients without CTG18.1 expansions) will enable researchers to further dissect and understand CTG18.1-specific molecular signatures of dysregulation from more generalised downstream features of disease common to all genetic subtypes.

### 6.6 Future therapies for FECD

The management of end-stage FECD has been revolutionized in developed countries by the introduction of endothelial keratoplasty that can rapidly and effectively restore vision. Unfortunately, this requires an ample supply of corneal tissue of which there is a global shortage (Gain et al., 2016). To address these concerns, investigators have started to assess the use of alternative approaches towards the treatment of FECD. Use of agents such as Rho kinase inhibitors has shown the ability to reduce central corneal edema (Koizumi et al., 2013; Okumura et al., 2013). Cell-based approaches that utilize injected cultured corneal endothelial cells [reviewed in (Ong Tone et al., 2020)] have shown the ability to improve the corneal clarity of patients with FECD for up to 3 years (Kinoshita et al., 2018). As these technologies progress, they will enable surgeons to remove the diseased corneal endothelial cells. While these approaches provide hope for the future, there are a number of obstacles to overcome before they can be utilized on a routine basis.

An obvious, yet underappreciated way to treat FECD is to have in place measures that detect the disease at an early stage. Prevention of end-stage disease may have the benefits of avoiding surgical risks, maintaining good vision in patients with mild to moderate disease who do not meet a surgical threshold, and redirecting a limited corneal donor supply to other areas of need. Developing a specific therapy that can be delivered easily, preferably topically, at a pre-symptomatic stage of the disease and assessing the safety and efficacy through clinical trials are challenges that must be addressed. Although the clinical identification of mild guttae can be achieved with routine slit lamp bio-microscopy, widely available clinical testing to differentiate CTG18.1 expansion-mediated FECD from other genetic variants does not exist. Evaluating disease progression in clinical trials will not be an easy task. Due to the slowly progressive nature of FECD, large and lengthy trials will be necessary to differentiate changes in the rate of progression between study groups. In addition to standard psychometric measurements of visual function (visual acuity, contract sensitivity, and glare), outcome metrics are likely to entail both anatomical variables that reflect the density of endothelial cells and guttae, and functional variables that relate to Na^+^/K^+^ ATPase pump function in corneal endothelial cells. Commonly used in vivo imaging of the corneal endothelial layer may be beneficial in some instances, but it also has limitations as the microscopic endothelial images suffer from sampling errors due to extreme variations in the density of guttae from the center to the periphery of the cornea. Better methods to assess anatomic disease severity need to be developed in order to efficiently identify individual guttae and cells so that accurate measurements of their density across the whole posterior corneal surface can occur. For functional testing, Scheimpflug imaging of the cornea may be useful to identify and classify stages of edema due to endothelial dysfunction (Sun et al., 2019). This type of imaging provides reproducible topography of corneal thickness across the majority of the cornea enabling mapping of thickness variation with resolution on the order of several microns (Patel et al., 2020). Studies utilizing Scheimpflug imaging have not yet been performed in cohorts of genotyped FECD patients, with and without CTG18.1 expansion or correlated with CTG18.1 length or zygosity status. If future phenotype-genotype correlations are found to emerge from use of this imaging modality, future application of this technology will likely aid the advancement of diagnostic and therapeutic approaches.

## 7 Concluding remarks

Over the past decade, significant progress has been made in defining the association between CTG18.1 expansion and FECD, in addition to identifying potential mechanisms that may underlie the disease. It is conceivable that no single mechanism but a combination of the mechanisms described herein and ones yet to be identified collectively drive FECD pathogenesis. Continued research over the coming years is necessary to increase our understanding of how these mechanisms contribute to disease pathophysiology. Development of animal models that recapitulate the disease phenotype and new imaging modalities that diagnose disease at an earlier stage are also anticipated to help identify and confirm novel therapeutic strategies. This will not be trivial given the possibility that multiple molecular drivers may be involved in disease formation. However, as our knowledge of CTG18.1 expansion-mediated FECD pathophysiology continues to advance, paralogous and increasingly promising avenues of research are anticipated that should promote development of novel preventative therapies.

## Author contributions

MPF, EDW, KHB, NB, ANS, NJHT, SJT and AED – literature searches, compilation of figures and tables, writing and editing of the manuscript.

## Declaration of competing interest

MPF, EDW, KHB, NB, ANS, NJHT, SJT – no conflicts of interest.

AED is a member of Triplet Therapeutics Scientific Advisory board. Research conducted in AED's laboratory is partly funded by ProQR Therapeutics.

## References

[bib1] Afshari N.A., Igo R.P., Morris N.J., Stambolian D., Sharma S., Pulagam V.L., Dunn S., Stamler J.F., Truitt B.J., Rimmler J., Kuot A., Croasdale C.R., Qin X., Burdon K.P., Riazuddin S.A., Mills R., Klebe S., Minear M.A., Zhao J., Balajonda E., Rosenwasser G.O., Baratz K.H., Mootha V.V., Patel S.V., Gregory S.G., Bailey-Wilson J.E., Price M.O., Price F.W., Craig J.E., Fingert J.H., Gottsch J.D., Aldave A.J., Klintworth G.K., Lass J.H., Li Y.J., Iyengar S.K. (2017). Genome-wide association study identifies three novel loci in Fuchs endothelial corneal dystrophy. Nat. Commun..

[bib2] Afshari N.A., Pittard A.B., Siddiqui A., Klintworth G.K. (2006). Clinical study of Fuchs corneal endothelial dystrophy leading to penetrating keratoplasty: a 30-year experience. Arch. Ophthalmol..

[bib3] Amiel J., Rio M., de Pontual L., Redon R., Malan V., Boddaert N., Plouin P., Carter N.P., Lyonnet S., Munnich A., Colleaux L. (2007). Mutations in TCF4, encoding a class I basic helix-loop-helix transcription factor, are responsible for Pitt-Hopkins syndrome, a severe epileptic encephalopathy associated with autonomic dysfunction. Am. J. Hum. Genet..

[bib4] Angelbello A.J., DeFeo M.E., Glinkerman C.M., Boger D.L., Disney M.D. (2020). Precise targeted cleavage of a r(CUG) repeat expansion in cells by using a small-molecule-deglycobleomycin conjugate. ACS Chem. Biol..

[bib5] Angelbello A.J., Rzuczek S.G., McKee K.K., Chen J.L., Olafson H., Cameron M.D., Moss W.N., Wang E.T., Disney M.D. (2019). Precise small-molecule cleavage of an r(CUG) repeat expansion in a myotonic dystrophy mouse model. Proc. Natl. Acad. Sci. U. S. A..

[bib6] Anvret M., Ahlberg G., Grandell U., Hedberg B., Johnson K., Edstrom L. (1993). Larger expansions of the CTG repeat in muscle compared to lymphocytes from patients with myotonic dystrophy. Hum. Mol. Genet..

[bib7] Ardui S., Ameur A., Vermeesch J.R., Hestand M.S. (2018). Single molecule real-time (SMRT) sequencing comes of age: applications and utilities for medical diagnostics. Nucleic Acids Res..

[bib8] Ash P.E., Bieniek K.F., Gendron T.F., Caulfield T., Lin W.L., Dejesus-Hernandez M., van Blitterswijk M.M., Jansen-West K., Paul J.W., Rademakers R., Boylan K.B., Dickson D.W., Petrucelli L. (2013). Unconventional translation of C9ORF72 GGGGCC expansion generates insoluble polypeptides specific to c9FTD/ALS. Neuron.

[bib9] Ayhan F., Perez B.A., Shorrock H.K., Zu T., Banez-Coronel M., Reid T., Furuya H., Clark H.B., Troncoso J.C., Ross C.A., Subramony S.H., Ashizawa T., Wang E.T., Yachnis A.T., Ranum L.P. (2018). SCA8 RAN polySer protein preferentially accumulates in white matter regions and is regulated by eIF3F. EMBO J..

[bib10] Babacic H., Mehta A., Merkel O., Schoser B. (2019). CRISPR-cas gene-editing as plausible treatment of neuromuscular and nucleotide-repeat-expansion diseases: a systematic review. PloS One.

[bib11] Balendra R., Isaacs A.M. (2018). C9orf72-mediated ALS and FTD: multiple pathways to disease. Nat. Rev. Neurol..

[bib12] Banez-Coronel M., Ayhan F., Tarabochia A.D., Zu T., Perez B.A., Tusi S.K., Pletnikova O., Borchelt D.R., Ross C.A., Margolis R.L., Yachnis A.T., Troncoso J.C., Ranum L.P. (2015). RAN translation in Huntington disease. Neuron.

[bib13] Baratz K.H., McLaren J.W., Maguire L.J., Patel S.V. (2012). Corneal haze determined by confocal microscopy 2 years after Descemet stripping with endothelial keratoplasty for Fuchs corneal dystrophy. Arch. Ophthalmol..

[bib14] Baratz K.H., Tosakulwong N., Ryu E., Brown W.L., Branham K., Chen W., Tran K.D., Schmid-Kubista K.E., Heckenlively J.R., Swaroop A., Abecasis G., Bailey K.R., Edwards A.O. (2010). E2-2 protein and Fuchs's corneal dystrophy. N. Engl. J. Med..

[bib15] Bayly R., Chuen L., Currie R.A., Hyndman B.D., Casselman R., Blobel G.A., LeBrun D.P. (2004). E2A-PBX1 interacts directly with the KIX domain of CBP/p300 in the induction of proliferation in primary hematopoietic cells. J. Biol. Chem..

[bib16] Bidichandani S.I., Ashizawa T., Patel P.I. (1998). The GAA triplet-repeat expansion in Friedreich ataxia interferes with transcription and may be associated with an unusual DNA structure. Am. J. Hum. Genet..

[bib17] Biswas S., Munier F.L., Yardley J., Hart-Holden N., Perveen R., Cousin P., Sutphin J.E., Noble B., Batterbury M., Kielty C., Hackett A., Bonshek R., Ridgway A., McLeod D., Sheffield V.C., Stone E.M., Schorderet D.F., Black G.C. (2001). Missense mutations in COL8A2, the gene encoding the alpha2 chain of type VIII collagen, cause two forms of corneal endothelial dystrophy. Hum. Mol. Genet..

[bib18] Blake D.J., Forrest M., Chapman R.M., Tinsley C.L., O'Donovan M.C., Owen M.J. (2010). TCF4, schizophrenia, and pitt-hopkins syndrome. Schizophr. Bull..

[bib19] Blanluet M., Masliah-Planchon J., Giurgea I., Bielle F., Girard E., Andrianteranagna M., Clemenceau S., Bourneix C., Burglen L., Doummar D., Rapinat A., Oumoussa B.M., Ayrault O., Pouponnot C., Gentien D., Pierron G., Delattre O., Doz F., Bourdeaut F. (2019). SHH medulloblastoma in a young adult with a TCF4 germline pathogenic variation. Acta Neuropathol..

[bib20] Boeynaems S., Bogaert E., Michiels E., Gijselinck I., Sieben A., Jovicic A., De Baets G., Scheveneels W., Steyaert J., Cuijt I., Verstrepen K.J., Callaerts P., Rousseau F., Schymkowitz J., Cruts M., Van Broeckhoven C., Van Damme P., Gitler A.D., Robberecht W., Van Den Bosch L. (2016). Drosophila screen connects nuclear transport genes to DPR pathology in c9ALS/FTD. Sci. Rep..

[bib21] Bonanno J.A. (2003). Identity and regulation of ion transport mechanisms in the corneal endothelium. Prog. Retin. Eye Res..

[bib22] Bonanno J.A. (2012). Molecular mechanisms underlying the corneal endothelial pump. Exp. Eye Res..

[bib23] Botta A., Vallo L., Rinaldi F., Bonifazi E., Amati F., Biancolella M., Gambardella S., Mancinelli E., Angelini C., Meola G., Novelli G. (2007). Gene expression analysis in myotonic dystrophy: indications for a common molecular pathogenic pathway in DM1 and DM2. Gene Expr..

[bib24] Bourne W.M. (1985). Fuch's Corneal Dystrophy.

[bib25] Braz S.O., Acquaire J., Gourdon G., Gomes-Pereira M. (2018). Of mice and men: advances in the understanding of neuromuscular aspects of myotonic dystrophy. Front. Neurol..

[bib26] Brejchova K., Dudakova L., Skalicka P., Dobrovolny R., Masek P., Putzova M., Moosajee M., Tuft S.J., Davidson A.E., Liskova P. (2019). IPSC-derived corneal endothelial-like cells act as an appropriate model system to assess the impact of SLC4A11 variants on pre-mRNA splicing. Invest. Ophthalmol. Vis. Sci..

[bib27] Breschel T.S., McInnis M.G., Margolis R.L., Sirugo G., Corneliussen B., Simpson S.G., McMahon F.J., MacKinnon D.F., Xu J.F., Pleasant N., Huo Y., Ashworth R.G., Grundstrom C., Grundstrom T., Kidd K.K., DePaulo J.R., Ross C.A. (1997). A novel, heritable, expanding CTG repeat in an intron of the SEF2-1 gene on chromosome 18q21.1. Hum. Mol. Genet..

[bib28] Brockschmidt A., Todt U., Ryu S., Hoischen A., Landwehr C., Birnbaum S., Frenck W., Radlwimmer B., Lichter P., Engels H., Driever W., Kubisch C., Weber R.G. (2007). Severe mental retardation with breathing abnormalities (Pitt-Hopkins syndrome) is caused by haploinsufficiency of the neuronal bHLH transcription factor TCF4. Hum. Mol. Genet..

[bib29] Brook J.D., McCurrach M.E., Harley H.G., Buckler A.J., Church D., Aburatani H., Hunter K., Stanton V.P., Thirion J.P., Hudson T. (1992). Molecular basis of myotonic dystrophy: expansion of a trinucleotide (CTG) repeat at the 3' end of a transcript encoding a protein kinase family member. Cell.

[bib30] Browne S.E., Beal M.F. (2006). Oxidative damage in Huntington's disease pathogenesis. Antioxidants Redox Signal..

[bib31] Buddi R., Lin B., Atilano S.R., Zorapapel N.C., Kenney M.C., Brown D.J. (2002). Evidence of oxidative stress in human corneal diseases. J. Histochem. Cytochem..

[bib32] Chakraborty M., Selma-Soriano E., Magny E., Couso J.P., Perez-Alonso M., Charlet-Berguerand N., Artero R., Llamusi B. (2015). Pentamidine rescues contractility and rhythmicity in a Drosophila model of myotonic dystrophy heart dysfunction. Dis Model Mech.

[bib33] Chamberlain C.M., Ranum L.P. (2012). Mouse model of muscleblind-like 1 overexpression: skeletal muscle effects and therapeutic promise. Hum. Mol. Genet..

[bib34] Chatterjee N., Lin Y., Santillan B.A., Yotnda P., Wilson J.H. (2015). Environmental stress induces trinucleotide repeat mutagenesis in human cells. Proc. Natl. Acad. Sci. U. S. A..

[bib35] Chen E.S., Terry M.A., Shamie N., Phillips P.M., Friend D.J., McLeod S.D. (2008). Descemet-stripping automated endothelial keratoplasty: insertion using a novel 40/60 underfold technique for preservation of donor endothelium. Cornea.

[bib36] Chen P., Chen J.Z., Shao C.Y., Li C.Y., Zhang Y.D., Lu W.J., Fu Y., Gu P., Fan X. (2015). Treatment with retinoic acid and lens epithelial cell-conditioned medium in vitro directed the differentiation of pluripotent stem cells towards corneal endothelial cell-like cells. Exp Ther Med.

[bib37] Cheng A.C., Pang C.P., Leung A.T., Chua J.K., Fan D.S., Lam D.S. (2000). The association between cigarette smoking and ocular diseases. Hong Kong medical journal = Xianggang yi xue za zhi.

[bib38] Christo C.G., van Rooij J., Geerards A.J., Remeijer L., Beekhuis W.H. (2001). Suture-related complications following keratoplasty: a 5-year retrospective study. Cornea.

[bib39] Ciosi M., Maxwell A., Cumming S.A., Hensman Moss D.J., Alshammari A.M., Flower M.D., Durr A., Leavitt B.R., Roos R.A.C., Holmans P., Jones L., Langbehn D.R., Kwak S., Tabrizi S.J., Monckton D.G. (2019). A genetic association study of glutamine-encoding DNA sequence structures, somatic CAG expansion, and DNA repair gene variants, with Huntington disease clinical outcomes. EBioMedicine.

[bib40] Consortium G.M. (2019). CAG repeat not polyglutamine length determines timing of huntington's disease onset. Cell.

[bib41] Consortium G.M. (2015). Identification of genetic factors that modify clinical onset of huntington's disease. Cell.

[bib42] Corneliussen B., Thornell A., Hallberg B., Grundstrom T. (1991). Helix-loop-helix transcriptional activators bind to a sequence in glucocorticoid response elements of retrovirus enhancers. J. Virol..

[bib43] Cotticelli M.G., Crabbe A.M., Wilson R.B., Shchepinov M.S. (2013). Insights into the role of oxidative stress in the pathology of Friedreich ataxia using peroxidation resistant polyunsaturated fatty acids. Redox Biol.

[bib44] Cristofani R., Crippa V., Vezzoli G., Rusmini P., Galbiati M., Cicardi M.E., Meroni M., Ferrari V., Tedesco B., Piccolella M., Messi E., Carra S., Poletti A. (2018). The small heat shock protein B8 (HSPB8) efficiently removes aggregating species of dipeptides produced in C9ORF72-related neurodegenerative diseases. Cell Stress Chaperones.

[bib45] Cross H.E., Maumenee A.E., Cantolino S.J. (1971). Inheritance of Fuchs' endothelial dystrophy. Arch. Ophthalmol..

[bib46] Cumming S.A., Hamilton M.J., Robb Y., Gregory H., McWilliam C., Cooper A., Adam B., McGhie J., Hamilton G., Herzyk P., Tschannen M.R., Worthey E., Petty R., Ballantyne B., Warner J., Farrugia M.E., Longman C., Monckton D.G. (2018). De novo repeat interruptions are associated with reduced somatic instability and mild or absent clinical features in myotonic dystrophy type 1. Eur. J. Hum. Genet..

[bib47] Davidson A.E., Liskova P., Evans C.J., Dudakova L., Noskova L., Pontikos N., Hartmannova H., Hodanova K., Stranecky V., Kozmik Z., Levis H.J., Idigo N., Sasai N., Maher G.J., Bellingham J., Veli N., Ebenezer N.D., Cheetham M.E., Daniels J.T., Thaung C.M., Jirsova K., Plagnol V., Filipec M., Kmoch S., Tuft S.J., Hardcastle A.J. (2016). Autosomal-dominant corneal endothelial dystrophies CHED1 and PPCD1 are allelic disorders caused by non-coding mutations in the promoter of OVOL2. Am. J. Hum. Genet..

[bib48] De Antonio M., Dogan C., Hamroun D., Mati M., Zerrouki S., Eymard B., Katsahian S., Bassez G. (2016). Unravelling the myotonic dystrophy type 1 clinical spectrum: a systematic registry-based study with implications for disease classification. Rev. Neurol. (Paris).

[bib49] DeJesus-Hernandez M., Mackenzie I.R., Boeve B.F., Boxer A.L., Baker M., Rutherford N.J., Nicholson A.M., Finch N.A., Flynn H., Adamson J., Kouri N., Wojtas A., Sengdy P., Hsiung G.Y., Karydas A., Seeley W.W., Josephs K.A., Coppola G., Geschwind D.H., Wszolek Z.K., Feldman H., Knopman D.S., Petersen R.C., Miller B.L., Dickson D.W., Boylan K.B., Graff-Radford N.R., Rademakers R. (2011). Expanded GGGGCC hexanucleotide repeat in noncoding region of C9ORF72 causes chromosome 9p-linked FTD and ALS. Neuron.

[bib50] Del-Favero J., Gestel S.V., Borglum A.D., Muir W., Ewald H., Mors O., Ivezic S., Oruc L., Adolfsson R., Blackwood D., Kruse T., Mendlewicz J., Schalling M., Van Broeckhoven C. (2002). European combined analysis of the CTG18.1 and the ERDA1 CAG/CTG repeats in bipolar disorder. Eur. J. Hum. Genet..

[bib51] Dogan C., De Antonio M., Hamroun D., Varet H., Fabbro M., Rougier F., Amarof K., Arne Bes M.C., Bedat-Millet A.L., Behin A., Bellance R., Bouhour F., Boutte C., Boyer F., Campana-Salort E., Chapon F., Cintas P., Desnuelle C., Deschamps R., Drouin-Garraud V., Ferrer X., Gervais-Bernard H., Ghorab K., Laforet P., Magot A., Magy L., Menard D., Minot M.C., Nadaj-Pakleza A., Pellieux S., Pereon Y., Preudhomme M., Pouget J., Sacconi S., Sole G., Stojkovich T., Tiffreau V., Urtizberea A., Vial C., Zagnoli F., Caranhac G., Bourlier C., Riviere G., Geille A., Gherardi R.K., Eymard B., Puymirat J., Katsahian S., Bassez G. (2016). Gender as a modifying factor influencing myotonic dystrophy type 1 phenotype severity and mortality: a nationwide multiple databases cross-sectional observational study. PloS One.

[bib52] Du J., Aleff R.A., Soragni E., Kalari K., Nie J., Tang X., Davila J., Kocher J.P., Patel S.V., Gottesfeld J.M., Baratz K.H., Wieben E.D. (2015). RNA toxicity and missplicing in the common eye disease fuchs endothelial corneal dystrophy. J. Biol. Chem..

[bib53] Ebbert M.T.W., Farrugia S.L., Sens J.P., Jansen-West K., Gendron T.F., Prudencio M., McLaughlin I.J., Bowman B., Seetin M., DeJesus-Hernandez M., Jackson J., Brown P.H., Dickson D.W., van Blitterswijk M., Rademakers R., Petrucelli L., Fryer J.D. (2018). Long-read sequencing across the C9orf72 'GGGGCC' repeat expansion: implications for clinical use and genetic discovery efforts in human disease. Mol. Neurodegener..

[bib54] Eghrari A.O., Gottsch J.D. (2010). Fuchs' corneal dystrophy. Expet Rev. Ophthalmol..

[bib55] Eghrari A.O., Vahedi S., Afshari N.A., Riazuddin S.A., Gottsch J.D. (2017). CTG18.1 expansion in TCF4 among african Americans with fuchs' corneal dystrophy. Invest. Ophthalmol. Vis. Sci..

[bib56] Eghrari A.O., Vasanth S., Wang J., Vahedi F., Riazuddin S.A., Gottsch J.D. (2017). CTG18.1 expansion in TCF4 increases likelihood of transplantation in fuchs corneal dystrophy. Cornea.

[bib57] Eichler E.E., Holden J.J., Popovich B.W., Reiss A.L., Snow K., Thibodeau S.N., Richards C.S., Ward P.A., Nelson D.L. (1994). Length of uninterrupted CGG repeats determines instability in the FMR1 gene. Nat. Genet..

[bib58] El-Kenawy A., Benarba B., Neves A.F., de Araujo T.G., Tan B.L., Gouri A. (2019). Gene surgery: potential applications for human diseases. EXCLI J.

[bib59] Ellenberger T., Fass D., Arnaud M., Harrison S.C. (1994). Crystal structure of transcription factor E47: E-box recognition by a basic region helix-loop-helix dimer. Genes Dev..

[bib60] Eye-Bank (2019). Eye-Bank Association of America. 2018 Eye Banking Statistical Report.

[bib61] Foiry L., Dong L., Savouret C., Hubert L., te Riele H., Junien C., Gourdon G. (2006). Msh3 is a limiting factor in the formation of intergenerational CTG expansions in DM1 transgenic mice. Hum. Genet..

[bib62] Foja S., Luther M., Hoffmann K., Rupprecht A., Gruenauer-Kloevekorn C. (2017). CTG18.1 repeat expansion may reduce TCF4 gene expression in corneal endothelial cells of German patients with Fuchs' dystrophy. Graefes Arch. Clin. Exp. Ophthalmol..

[bib63] Forrest M., Chapman R.M., Doyle A.M., Tinsley C.L., Waite A., Blake D.J. (2012). Functional analysis of TCF4 missense mutations that cause Pitt-Hopkins syndrome. Hum. Mutat..

[bib64] Forrest M.P., Hill M.J., Kavanagh D.H., Tansey K.E., Waite A.J., Blake D.J. (2018). The psychiatric risk gene transcription factor 4 (TCF4) regulates neurodevelopmental pathways associated with schizophrenia, autism, and intellectual disability. Schizophr. Bull..

[bib65] Forrest M.P., Hill M.J., Quantock A.J., Martin-Rendon E., Blake D.J. (2014). The emerging roles of TCF4 in disease and development. Trends Mol. Med..

[bib66] Forrest M.P., Waite A.J., Martin-Rendon E., Blake D.J. (2013). Knockdown of human TCF4 affects multiple signaling pathways involved in cell survival, epithelial to mesenchymal transition and neuronal differentiation. PloS One.

[bib67] Forstot S.L., Abel R., Binder P.S. (1975). Bacterial endophthalmitis following suture removal after penetrating keratoplasty. Am. J. Ophthalmol..

[bib68] Foulks G.N. (1981). Treatment of recurrent corneal erosion and corneal edema with topical osmotic colloidal solution. Ophthalmology.

[bib69] Gabrieli T., Sharim H., Fridman D., Arbib N., Michaeli Y., Ebenstein Y. (2018). Selective nanopore sequencing of human BRCA1 by Cas9-assisted targeting of chromosome segments (CATCH). Nucleic Acids Res..

[bib70] Gain P., Jullienne R., He Z., Aldossary M., Acquart S., Cognasse F., Thuret G. (2016). Global survey of corneal transplantation and eye banking. JAMA Ophthalmol.

[bib71] Gipson T.A., Neueder A., Wexler N.S., Bates G.P., Housman D. (2013). Aberrantly spliced HTT, a new player in Huntington's disease pathogenesis. RNA Biol..

[bib72] Goar E.L. (1933). Dystrophy of the corneal endothelium (cornea guttata), with report of a histologic examination. Trans. Am. Ophthalmol. Soc..

[bib73] Goldfarb A.N., Lewandowska K., Pennell C.A. (1998). Identification of a highly conserved module in E proteins required for in vivo helix-loop-helix dimerization. J. Biol. Chem..

[bib74] Gomes-Pereira M., Monckton D.G. (2004). Mouse tissue culture models of unstable triplet repeats. Methods Mol. Biol..

[bib75] Grayson M. (1983). Diseases of the Cornea.

[bib76] Greb-Markiewicz B., Kazana W., Zarebski M., Ozyhar A. (2019). The subcellular localization of bHLH transcription factor TCF4 is mediated by multiple nuclear localization and nuclear export signals. Sci. Rep..

[bib77] Group H.s.D.C.R. (1993). A novel gene containing a trinucleotide repeat that is expanded and unstable on Huntington's disease chromosomes. The Huntington's Disease Collaborative Research Group. Cell.

[bib78] Gupta R., Kumawat B.L., Paliwal P., Tandon R., Sharma N., Sen S., Kashyap S., Nag T.C., Vajpayee R.B., Sharma A. (2015). Association of ZEB1 and TCF4 rs613872 changes with late onset Fuchs endothelial corneal dystrophy in patients from northern India. Mol. Vis..

[bib79] Haeusler A.R., Donnelly C.J., Periz G., Simko E.A., Shaw P.G., Kim M.S., Maragakis N.J., Troncoso J.C., Pandey A., Sattler R., Rothstein J.D., Wang J. (2014). C9orf72 nucleotide repeat structures initiate molecular cascades of disease. Nature.

[bib80] Hafford-Tear N.J., Tsai Y.C., Sadan A.N., Sanchez-Pintado B., Zarouchlioti C., Maher G.J., Liskova P., Tuft S.J., Hardcastle A.J., Clark T.A., Davidson A.E. (2019). CRISPR/Cas9-targeted enrichment and long-read sequencing of the Fuchs endothelial corneal dystrophy-associated TCF4 triplet repeat. Genet. Med..

[bib81] Hardwick S.A., Joglekar A., Flicek P., Frankish A., Tilgner H.U. (2019). Getting the entire message: progress in isoform sequencing. Front. Genet..

[bib82] Harper P.S., Harley H.G., Reardon W., Shaw D.J. (1992). Anticipation in myotonic dystrophy: new light on an old problem. Am. J. Hum. Genet..

[bib83] Hatou S., Shimmura S. (2019). Review: corneal endothelial cell derivation methods from ES/iPS cells. Inflamm. Regen..

[bib84] Hellwig M., Lauffer M.C., Bockmayr M., Spohn M., Merk D.J., Harrison L., Ahlfeld J., Kitowski A., Neumann J.E., Ohli J., Holdhof D., Niesen J., Schoof M., Kool M., Kraus C., Zweier C., Holmberg D., Schuller U. (2019). TCF4 (E2-2) harbors tumor suppressive functions in SHH medulloblastoma. Acta Neuropathol..

[bib85] Henthorn P., Kiledjian M., Kadesch T. (1990). Two distinct transcription factors that bind the immunoglobulin enhancer microE5/kappa 2 motif. Science.

[bib86] Higa A., Sakai H., Sawaguchi S., Iwase A., Tomidokoro A., Amano S., Araie M. (2011). Prevalence of and risk factors for cornea guttata in a population-based study in a southwestern island of Japan: the Kumejima study. Arch. Ophthalmol..

[bib87] Hoijer I., Johansson J., Gudmundsson S., Chin C.-S., Bunikis I., Haggqvist S., Emmanouilidou A., Wilbe M., den Hoed M., Bondeson M.-L., Feuk L., Gyllensten U., Ameur A. (2020). Amplification-free Long Read Sequencing Reveals Unforeseen CRISPR-Cas9 Off-Target Activity.

[bib88] Hoijer I., Tsai Y.C., Clark T.A., Kotturi P., Dahl N., Stattin E.L., Bondeson M.L., Feuk L., Gyllensten U., Ameur A. (2018). Detailed analysis of HTT repeat elements in human blood using targeted amplification-free long-read sequencing. Hum. Mutat..

[bib89] Hrckulak D., Kolar M., Strnad H., Korinek V. (2016). TCF/LEF transcription factors: an update from the internet resources. Cancers (Basel).

[bib90] Hu J., Rong Z., Gong X., Zhou Z., Sharma V.K., Xing C., Watts J.K., Corey D.R., Mootha V.V. (2018). Oligonucleotides targeting TCF4 triplet repeat expansion inhibit RNA foci and mis-splicing in Fuchs' dystrophy. Hum. Mol. Genet..

[bib91] Hu J., Shen X., Rigo F., Prakash T.P., Mootha V.V., Corey D.R. (2019). Duplex RNAs and ss-siRNAs block RNA foci associated with fuchs' endothelial corneal dystrophy. Nucleic Acid Therapeut..

[bib92] Huichalaf C., Sakai K., Jin B., Jones K., Wang G.L., Schoser B., Schneider-Gold C., Sarkar P., Pereira-Smith O.M., Timchenko N., Timchenko L. (2010). Expansion of CUG RNA repeats causes stress and inhibition of translation in myotonic dystrophy 1 (DM1) cells. Faseb. J..

[bib93] Ihara Y., Mori A., Hayabara T., Namba R., Nobukuni K., Sato K., Miyata S., Edamatsu R., Liu J., Kawai M. (1995). Free radicals, lipid peroxides and antioxidants in blood of patients with myotonic dystrophy. J. Neurol..

[bib94] Jain A., Vale R.D. (2017). RNA phase transitions in repeat expansion disorders. Nature.

[bib95] Jansen G., Willems P., Coerwinkel M., Nillesen W., Smeets H., Vits L., Howeler C., Brunner H., Wieringa B. (1994). Gonosomal mosaicism in myotonic dystrophy patients: involvement of mitotic events in (CTG)n repeat variation and selection against extreme expansion in sperm. Am. J. Hum. Genet..

[bib96] Jenquin J.R., Coonrod L.A., Silverglate Q.A., Pellitier N.A., Hale M.A., Xia G., Nakamori M., Berglund J.A. (2018). Furamidine rescues myotonic dystrophy type I associated mis-splicing through multiple mechanisms. ACS Chem. Biol..

[bib97] Jenquin J.R., Yang H., Huigens R.W., Nakamori M., Berglund J.A. (2019). Combination treatment of erythromycin and furamidine provides additive and synergistic rescue of mis-splicing in myotonic dystrophy type 1 models. ACS Pharmacol Transl Sci.

[bib98] Jinek M., Chylinski K., Fonfara I., Hauer M., Doudna J.A., Charpentier E. (2012). A programmable dual-RNA-guided DNA endonuclease in adaptive bacterial immunity. Science.

[bib99] Jovicic A., Mertens J., Boeynaems S., Bogaert E., Chai N., Yamada S.B., Paul J.W., Sun S., Herdy J.R., Bieri G., Kramer N.J., Gage F.H., Van Den Bosch L., Robberecht W., Gitler A.D. (2015). Modifiers of C9orf72 dipeptide repeat toxicity connect nucleocytoplasmic transport defects to FTD/ALS. Nat. Neurosci..

[bib100] Joyce N.C. (2012). Proliferative capacity of corneal endothelial cells. Exp. Eye Res..

[bib101] Joyce N.C., Harris D.L., Mello D.M. (2002). Mechanisms of mitotic inhibition in corneal endothelium: contact inhibition and TGF-beta2. Invest. Ophthalmol. Vis. Sci..

[bib102] Jurkunas U.V. (2018). Fuchs endothelial corneal dystrophy through the prism of oxidative stress. Cornea.

[bib103] Jurkunas U.V., Bitar M.S., Funaki T., Azizi B. (2010). Evidence of oxidative stress in the pathogenesis of fuchs endothelial corneal dystrophy. Am. J. Pathol..

[bib104] Kanadia R.N., Shin J., Yuan Y., Beattie S.G., Wheeler T.M., Thornton C.A., Swanson M.S. (2006). Reversal of RNA missplicing and myotonia after muscleblind overexpression in a mouse poly(CUG) model for myotonic dystrophy. Proc. Natl. Acad. Sci. U. S. A..

[bib105] Kinoshita M., Takahashi R., Hasegawa T., Komori T., Nagasawa R., Hirose K., Tanabe H. (1996). (CTG)n expansions in various tissues from a myotonic dystrophy patient. Muscle Nerve.

[bib106] Kinoshita S., Koizumi N., Ueno M., Okumura N., Imai K., Tanaka H., Yamamoto Y., Nakamura T., Inatomi T., Bush J., Toda M., Hagiya M., Yokota I., Teramukai S., Sotozono C., Hamuro J. (2018). Injection of cultured cells with a ROCK inhibitor for bullous keratopathy. N. Engl. J. Med..

[bib107] Kitagawa K., Kojima M., Sasaki H., Shui Y.B., Chew S.J., Cheng H.M., Ono M., Morikawa Y., Sasaki K. (2002). Prevalence of primary cornea guttata and morphology of corneal endothelium in aging Japanese and Singaporean subjects. Ophthalmic Res..

[bib108] Kohwi Y. (2004). Methods in Molecular Biology: Trinucleotide Repeat Protocols.

[bib109] Koizumi N., Okumura N., Ueno M., Nakagawa H., Hamuro J., Kinoshita S. (2013). Rho-associated kinase inhibitor eye drop treatment as a possible medical treatment for Fuchs corneal dystrophy. Cornea.

[bib110] Kovtun I.V., Liu Y., Bjoras M., Klungland A., Wilson S.H., McMurray C.T. (2007). OGG1 initiates age-dependent CAG trinucleotide expansion in somatic cells. Nature.

[bib111] Krachmer J.H., Purcell J.J., Young C.W., Bucher K.D. (1978). Corneal endothelial dystrophy. A study of 64 families. Arch. Ophthalmol..

[bib112] Krafchak C.M., Pawar H., Moroi S.E., Sugar A., Lichter P.R., Mackey D.A., Mian S., Nairus T., Elner V., Schteingart M.T., Downs C.A., Kijek T.G., Johnson J.M., Trager E.H., Rozsa F.W., Mandal M.N., Epstein M.P., Vollrath D., Ayyagari R., Boehnke M., Richards J.E. (2005). Mutations in TCF8 cause posterior polymorphous corneal dystrophy and ectopic expression of COL4A3 by corneal endothelial cells. Am. J. Hum. Genet..

[bib113] Kremer E.J., Pritchard M., Lynch M., Yu S., Holman K., Baker E., Warren S.T., Schlessinger D., Sutherland G.R., Richards R.I. (1991). Mapping of DNA instability at the fragile X to a trinucleotide repeat sequence p(CCG)n. Science.

[bib114] Kuot A., Hewitt A.W., Snibson G.R., Souzeau E., Mills R., Craig J.E., Burdon K.P., Sharma S. (2017). TGC repeat expansion in the TCF4 gene increases the risk of Fuchs' endothelial corneal dystrophy in Australian cases. PloS One.

[bib115] Kuyumcu-Martinez N.M., Wang G.S., Cooper T.A. (2007). Increased steady-state levels of CUGBP1 in myotonic dystrophy 1 are due to PKC-mediated hyperphosphorylation. Mol. Cell..

[bib116] Li J., Nakamori M., Matsumoto J., Murata A., Dohno C., Kiliszek A., Taylor K., Sobczak K., Nakatani K. (2018). A dimeric 2,9-Diamino-1,10-phenanthroline derivative improves alternative splicing in myotonic dystrophy Type 1 cell and mouse models. Chemistry (Weinheim an der Bergstrasse, Germany).

[bib117] Li S., Kim E., Bonanno J.A. (2016). Fluid transport by the cornea endothelium is dependent on buffering lactic acid efflux. Am. J. Physiol. Cell Physiol..

[bib118] Li Y.J., Minear M.A., Rimmler J., Zhao B., Balajonda E., Hauser M.A., Allingham R.R., Eghrari A.O., Riazuddin S.A., Katsanis N., Gottsch J.D., Gregory S.G., Klintworth G.K., Afshari N.A. (2011). Replication of TCF4 through association and linkage studies in late-onset Fuchs endothelial corneal dystrophy. PloS One.

[bib119] Lin X., Miller J.W., Mankodi A., Kanadia R.N., Yuan Y., Moxley R.T., Swanson M.S., Thornton C.A. (2006). Failure of MBNL1-dependent post-natal splicing transitions in myotonic dystrophy. Hum. Mol. Genet..

[bib120] Liquori C.L., Ricker K., Moseley M.L., Jacobsen J.F., Kress W., Naylor S.L., Day J.W., Ranum L.P. (2001). Myotonic dystrophy type 2 caused by a CCTG expansion in intron 1 of ZNF9. Science.

[bib121] Liskova P., Dudakova L., Evans C.J., Rojas Lopez K.E., Pontikos N., Athanasiou D., Jama H., Sach J., Skalicka P., Stranecky V., Kmoch S., Thaung C., Filipec M., Cheetham M.E., Davidson A.E., Tuft S.J., Hardcastle A.J. (2018). Ectopic GRHL2 expression due to non-coding mutations promotes cell state transition and causes posterior polymorphous corneal dystrophy 4. Am. J. Hum. Genet..

[bib122] Liu C., Miyajima T., Melangath G., Miyai T., Vasanth S., Deshpande N., Kumar V., Ong Tone S., Gupta R., Zhu S., Vojnovic D., Chen Y., Rogan E.G., Mondal B., Zahid M., Jurkunas U.V. (2020). Ultraviolet A light induces DNA damage and estrogen-DNA adducts in Fuchs endothelial corneal dystrophy causing females to be more affected. Proc. Natl. Acad. Sci. U. S. A..

[bib123] Liu C., Vojnovic D., Kochevar I.E., Jurkunas U.V. (2016). UV-A irradiation activates nrf2-regulated antioxidant defense and induces p53/caspase3-dependent apoptosis in corneal endothelial cells. Invest. Ophthalmol. Vis. Sci..

[bib124] Liu C.R., Chang C.R., Chern Y., Wang T.H., Hsieh W.C., Shen W.C., Chang C.Y., Chu I.C., Deng N., Cohen S.N., Cheng T.H. (2012). Spt4 is selectively required for transcription of extended trinucleotide repeats. Cell.

[bib125] Long A., Napierala J.S., Polak U., Hauser L., Koeppen A.H., Lynch D.R., Napierala M. (2017). Somatic instability of the expanded GAA repeats in Friedreich's ataxia. PloS One.

[bib126] Longo A., Guanga G.P., Rose R.B. (2008). Crystal structure of E47-NeuroD1/beta2 bHLH domain-DNA complex: heterodimer selectivity and DNA recognition. Biochemistry.

[bib127] Lopez Castel A., Nakamori M., Tome S., Chitayat D., Gourdon G., Thornton C.A., Pearson C.E. (2011). Expanded CTG repeat demarcates a boundary for abnormal CpG methylation in myotonic dystrophy patient tissues. Hum. Mol. Genet..

[bib128] Lorenzetti D.W., Uotila M.H., Parikh N., Kaufman H.E. (1967). Central cornea guttata. Incidence in the general population. Am. J. Ophthalmol..

[bib129] Luther M., Grunauer-Kloevekorn C., Weidle E., Passarge E., Rupprecht A., Hoffmann K., Foja S. (2016). [TGC repeats in intron 2 of the TCF4 gene have a good predictive power regarding to fuchs endothelial corneal dystrophy]. Klin Monbl Augenheilkd.

[bib130] Luu L.M., Nguyen L., Peng S., Lee J., Lee H.Y., Wong C.H., Hergenrother P.J., Chan H.Y., Zimmerman S.C. (2016). A potent inhibitor of protein sequestration by expanded triplet (CUG) repeats that shows phenotypic improvements in a Drosophila model of myotonic dystrophy. ChemMedChem.

[bib131] Maiuri T., Bowie L.E., Truant R. (2019). DNA repair signaling of huntingtin: the next link between late-onset neurodegenerative disease and oxidative DNA damage. DNA Cell Biol..

[bib132] Mannis M.J., Zadnik K., Miller M.R., Marquez M. (1997). Preoperative risk factors for surface disease after penetrating keratoplasty. Cornea.

[bib133] Mason A.G., Tome S., Simard J.P., Libby R.T., Bammler T.K., Beyer R.P., Morton A.J., Pearson C.E., La Spada A.R. (2014). Expression levels of DNA replication and repair genes predict regional somatic repeat instability in the brain but are not altered by polyglutamine disease protein expression or age. Hum. Mol. Genet..

[bib134] Massari M.E., Grant P.A., Pray-Grant M.G., Berger S.L., Workman J.L., Murre C. (1999). A conserved motif present in a class of helix-loop-helix proteins activates transcription by direct recruitment of the SAGA complex. Mol. Cell..

[bib135] Massari M.E., Murre C. (2000). Helix-loop-helix proteins: regulators of transcription in eucaryotic organisms. Mol. Cell Biol..

[bib136] Matloka M., Klein A.F., Rau F., Furling D. (2018). Cells of matter-in vitro models for myotonic dystrophy. Front. Neurol..

[bib137] Matthaei M., Sandhaeger H., Hermel M., Adler W., Jun A.S., Cursiefen C., Heindl L.M. (2017). Changing indications in penetrating keratoplasty: a systematic review of 34 Years of global reporting. Transplantation.

[bib138] McCabe K.L., Kunzevitzky N.J., Chiswell B.P., Xia X., Goldberg J.L., Lanza R. (2015). Efficient generation of human embryonic stem cell-derived corneal endothelial cells by directed differentiation. PloS One.

[bib139] McInnis M.G., Swift-Scanlanl T., Mahoney A.T., Vincent J., Verheyen G., Lan T.H., Oruc L., Riess O., Van Broeckhoven C., Chen H., Kennedy J.L., MacKinnon D.F., Margolis R.L., Simpson S.G., McMahon F.J., Gershon E., Nurnberger J., Reich T., DePaulo J.R., Ross C.A. (2000). Allelic distribution of CTG18.1 in Caucasian populations: association studies in bipolar disorder, schizophrenia, and ataxia. Mol. Psychiatr..

[bib140] McMurray C.T. (2010). Mechanisms of trinucleotide repeat instability during human development. Nat. Rev. Genet..

[bib141] Mehta J.S., Vithana E.N., Tan D.T., Yong V.H., Yam G.H., Law R.W., Chong W.G., Pang C.P., Aung T. (2008). Analysis of the posterior polymorphous corneal dystrophy 3 gene, TCF8, in late-onset Fuchs endothelial corneal dystrophy. Invest. Ophthalmol. Vis. Sci..

[bib142] Meyer R.F., Bobb K.C. (1980). Corneal epithelium in penetrating keratoplasty. Am. J. Ophthalmol..

[bib143] Mohan A., Goodwin M., Swanson M.S. (2014). RNA-protein interactions in unstable microsatellite diseases. Brain Res..

[bib144] Monckton D.G., Wong L.J., Ashizawa T., Caskey C.T. (1995). Somatic mosaicism, germline expansions, germline reversions and intergenerational reductions in myotonic dystrophy males: small pool PCR analyses. Hum. Mol. Genet..

[bib145] Mootha V.V., Gong X., Ku H.C., Xing C. (2014). Association and familial segregation of CTG18.1 trinucleotide repeat expansion of TCF4 gene in Fuchs' endothelial corneal dystrophy. Invest. Ophthalmol. Vis. Sci..

[bib146] Mootha V.V., Hansen B., Rong Z., Mammen P.P., Zhou Z., Xing C., Gong X. (2017). Fuchs' endothelial corneal dystrophy and RNA foci in patients with myotonic dystrophy. Invest. Ophthalmol. Vis. Sci..

[bib147] Mootha V.V., Hussain I., Cunnusamy K., Graham E., Gong X., Neelam S., Xing C., Kittler R., Petroll W.M. (2015). TCF4 triplet repeat expansion and nuclear RNA foci in fuchs' endothelial corneal dystrophy. Invest. Ophthalmol. Vis. Sci..

[bib148] Morales F., Couto J.M., Higham C.F., Hogg G., Cuenca P., Braida C., Wilson R.H., Adam B., del Valle G., Brian R., Sittenfeld M., Ashizawa T., Wilcox A., Wilcox D.E., Monckton D.G. (2012). Somatic instability of the expanded CTG triplet repeat in myotonic dystrophy type 1 is a heritable quantitative trait and modifier of disease severity. Hum. Mol. Genet..

[bib149] Morales F., Vasquez M., Santamaria C., Cuenca P., Corrales E., Monckton D.G. (2016). A polymorphism in the MSH3 mismatch repair gene is associated with the levels of somatic instability of the expanded CTG repeat in the blood DNA of myotonic dystrophy type 1 patients. DNA Repair.

[bib150] Mori K., Weng S.M., Arzberger T., May S., Rentzsch K., Kremmer E., Schmid B., Kretzschmar H.A., Cruts M., Van Broeckhoven C., Haass C., Edbauer D. (2013). The C9orf72 GGGGCC repeat is translated into aggregating dipeptide-repeat proteins in FTLD/ALS. Science.

[bib151] Mornet E., Chateau C., Hirst M.C., Thepot F., Taillandier A., Cibois O., Serre J.L. (1996). Analysis of germline variation at the FMR1 CGG repeat shows variation in the normal-premutated borderline range. Hum. Mol. Genet..

[bib152] Murre C., McCaw P.S., Baltimore D. (1989). A new DNA binding and dimerization motif in immunoglobulin enhancer binding, daughterless, MyoD, and myc proteins. Cell.

[bib153] Nakamori M., Panigrahi G.B., Lanni S., Gall-Duncan T., Hayakawa H., Tanaka H., Luo J., Otabe T., Li J., Sakata A., Caron M.C., Joshi N., Prasolava T., Chiang K., Masson J.Y., Wold M.S., Wang X., Lee M., Huddleston J., Munson K.M., Davidson S., Layeghifard M., Edward L.M., Gallon R., Santibanez-Koref M., Murata A., Takahashi M.P., Eichler E.E., Shlien A., Nakatani K., Mochizuki H., Pearson C.E. (2020). A slipped-CAG DNA-binding small molecule induces trinucleotide-repeat contractions in vivo. Nat. Genet..

[bib154] Nakamori M., Sobczak K., Puwanant A., Welle S., Eichinger K., Pandya S., Dekdebrun J., Heatwole C.R., McDermott M.P., Chen T., Cline M., Tawil R., Osborne R.J., Wheeler T.M., Swanson M.S., Moxley R.T., Thornton C.A. (2013). Splicing biomarkers of disease severity in myotonic dystrophy. Ann. Neurol..

[bib155] Nakano M., Okumura N., Nakagawa H., Koizumi N., Ikeda Y., Ueno M., Yoshii K., Adachi H., Aleff R.A., Butz M.L., Highsmith W.E., Tashiro K., Wieben E.D., Kinoshita S., Baratz K.H. (2015). Trinucleotide repeat expansion in the TCF4 gene in fuchs' endothelial corneal dystrophy in Japanese. Invest. Ophthalmol. Vis. Sci..

[bib156] Nanda G.G., Padhy B., Samal S., Das S., Alone D.P. (2014). Genetic association of TCF4 intronic polymorphisms, CTG18.1 and rs17089887, with Fuchs' endothelial corneal dystrophy in an Indian population. Invest. Ophthalmol. Vis. Sci..

[bib157] Nguyen L., Montrasio F., Pattamatta A., Tusi S.K., Bardhi O., Meyer K.D., Hayes L., Nakamura K., Banez-Coronel M., Coyne A., Guo S., Laboissonniere L.A., Gu Y., Narayanan S., Smith B., Nitsch R.M., Kankel M.W., Rushe M., Rothstein J., Zu T., Grimm J., Ranum L.P.W. (2020). Antibody therapy targeting RAN proteins rescues C9 ALS/FTD phenotypes in C9orf72 mouse model. Neuron.

[bib158] Niblock M., Smith B.N., Lee Y.B., Sardone V., Topp S., Troakes C., Al-Sarraj S., Leblond C.S., Dion P.A., Rouleau G.A., Shaw C.E., Gallo J.M. (2016). Retention of hexanucleotide repeat-containing intron in C9orf72 mRNA: implications for the pathogenesis of ALS/FTD. Acta Neuropathol Commun.

[bib159] O'Hearn E.E., Hwang H.S., Holmes S.E., Rudnicki D.D., Chung D.W., Seixas A.I., Cohen R.L., Ross C.A., Trojanowski J.Q., Pletnikova O., Troncoso J.C., Margolis R.L. (2015). Neuropathology and cellular pathogenesis of spinocerebellar ataxia type 12. Mov. Disord..

[bib160] Ohya K., Tachi N., Kon S., Kikuchi K., Chiba S. (1995). Somatic cell heterogeneity between DNA extracted from lymphocytes and skeletal muscle in congenital myotonic dystrophy. Jpn. J. Hum. Genet..

[bib161] Okumura N., Hayashi R., Nakano M., Tashiro K., Yoshii K., Aleff R., Butz M., Highsmith E.W., Wieben E.D., Fautsch M.P., Baratz K.H., Komori Y., Ueda E., Nakahara M., Weller J., Tourtas T., Schlotzer-Schrehardt U., Kruse F., Koizumi N. (2019). Association of rs613872 and trinucleotide repeat expansion in the TCF4 gene of German patients with fuchs endothelial corneal dystrophy. Cornea.

[bib162] Okumura N., Hayashi R., Nakano M., Yoshii K., Tashiro K., Sato T., Blake D.J., Aleff R., Butz M., Highsmith E.W., Wieben E.D., Fautsch M.P., Baratz K.H., Komori Y., Nakahara M., Tourtas T., Schlotzer-Schrehardt U., Kruse F., Koizumi N. (2019). Effect of trinucleotide repeat expansion on the expression of TCF4 mRNA in fuchs' endothelial corneal dystrophy. Invest. Ophthalmol. Vis. Sci..

[bib163] Okumura N., Koizumi N. (2020). Regeneration of the corneal endothelium. Curr. Eye Res..

[bib164] Okumura N., Koizumi N., Kay E.P., Ueno M., Sakamoto Y., Nakamura S., Hamuro J., Kinoshita S. (2013). The ROCK inhibitor eye drop accelerates corneal endothelium wound healing. Invest. Ophthalmol. Vis. Sci..

[bib165] Okumura N., Puangsricharern V., Jindasak R., Koizumi N., Komori Y., Ryousuke H., Nakahara M., Nakano M., Adachi H., Tashiro K., Yoshii K., Chantaren P., Ittiwut R., Shotelersuk V., Suphapeetiporn K. (2019). Trinucleotide repeat expansion in the transcription factor 4 (TCF4) gene in Thai patients with Fuchs endothelial corneal dystrophy. Eye.

[bib166] Oldak M., Ruszkowska E., Udziela M., Ozieblo D., Binczyk E., Sciezynska A., Ploski R., Szaflik J.P. (2015). Fuchs endothelial corneal dystrophy: strong association with rs613872 not paralleled by changes in corneal endothelial TCF4 mRNA level. BioMed Res. Int..

[bib167] Ong Tone S., Kocaba V., Böhm M., Wylegala A., White T.L., Jurkunas U.V. (2020). Fuchs endothelial corneal dystrophy: the vicious cycle of Fuchs pathogenesis. Prog. Retin. Eye Res..

[bib168] Onishi H., Kino Y., Morita T., Futai E., Sasagawa N., Ishiura S. (2008). MBNL1 associates with YB-1 in cytoplasmic stress granules. J. Neurosci. Res..

[bib169] Orr H.T., Zoghbi H.Y. (2007). Trinucleotide repeat disorders. Annu. Rev. Neurosci..

[bib170] Patel S.V. (2012). Graft survival and endothelial outcomes in the new era of endothelial keratoplasty. Exp. Eye Res..

[bib171] Patel S.V., Baratz K.H., Hodge D.O., Maguire L.J., McLaren J.W. (2009). The effect of corneal light scatter on vision after descemet stripping with endothelial keratoplasty. Arch. Ophthalmol..

[bib172] Patel S.V., Baratz K.H., Maguire L.J., Hodge D.O., McLaren J.W. (2012). Anterior corneal aberrations after Descemet's stripping endothelial keratoplasty for Fuchs' endothelial dystrophy. Ophthalmology.

[bib173] Patel S.V., Hodge D.O., Treichel E.J., Spiegel M.R., Baratz K.H. (2020). Repeatability of Scheimpflug tomography for assessing fuchs endothelial corneal dystrophy. Am. J. Ophthalmol..

[bib174] Paulson H. (2018). Repeat expansion diseases. Handb. Clin. Neurol..

[bib175] Peh G.S., Chng Z., Ang H.P., Cheng T.Y., Adnan K., Seah X.Y., George B.L., Toh K.P., Tan D.T., Yam G.H., Colman A., Mehta J.S. (2015). Propagation of human corneal endothelial cells: a novel dual media approach. Cell Transplant..

[bib176] Perbellini R., Greco S., Sarra-Ferraris G., Cardani R., Capogrossi M.C., Meola G., Martelli F. (2011). Dysregulation and cellular mislocalization of specific miRNAs in myotonic dystrophy type 1. Neuromuscul. Disord..

[bib177] Pham T.T., Yin J., Eid J.S., Adams E., Lam R., Turner S.W., Loomis E.W., Wang J.Y., Hagerman P.J., Hanes J.W. (2016). Single-locus enrichment without amplification for sequencing and direct detection of epigenetic modifications. Mol. Genet. Genom..

[bib178] Pinto B.S., Saxena T., Oliveira R., Mendez-Gomez H.R., Cleary J.D., Denes L.T., McConnell O., Arboleda J., Xia G., Swanson M.S., Wang E.T. (2017). Impeding transcription of expanded microsatellite repeats by deactivated Cas9. Mol. Cell..

[bib179] Price F.W., Price M.O. (2005). Descemet's stripping with endothelial keratoplasty in 50 eyes: a refractive neutral corneal transplant. J. Refract. Surg..

[bib180] Price F.W., Whitson W.E., Ahad K.A., Tavakkoli H. (1994). Suprachoroidal hemorrhage in penetrating keratoplasty. Ophthalmic Surg..

[bib181] Price M.O., Giebel A.W., Fairchild K.M., Price F.W. (2009). Descemet's membrane endothelial keratoplasty: prospective multicenter study of visual and refractive outcomes and endothelial survival. Ophthalmology.

[bib182] Pscherer A., Dorflinger U., Kirfel J., Gawlas K., Ruschoff J., Buettner R., Schule R. (1996). The helix-loop-helix transcription factor SEF-2 regulates the activity of a novel initiator element in the promoter of the human somatostatin receptor II gene. EMBO J..

[bib183] Purcell J.J., Krachmer J.H., Doughman D.J., Bourne W.M. (1982). Expulsive hemorrhage in penetrating keratoplasty. Ophthalmology.

[bib184] Raheem O., Olufemi S.E., Bachinski L.L., Vihola A., Sirito M., Holmlund-Hampf J., Haapasalo H., Li Y.P., Udd B., Krahe R. (2010). Mutant (CCTG)n expansion causes abnormal expression of zinc finger protein 9 (ZNF9) in myotonic dystrophy type 2. Am. J. Pathol..

[bib185] Ranganathan S., Harmison G.G., Meyertholen K., Pennuto M., Burnett B.G., Fischbeck K.H. (2009). Mitochondrial abnormalities in spinal and bulbar muscular atrophy. Hum. Mol. Genet..

[bib186] Rannals M.D., Maher B.J. (2017). Molecular mechanisms of transcription factor 4 in Pitt hopkins syndrome. Curr Genet Med Rep.

[bib187] Rao B.S., Tharigopala A., Rachapalli S.R., Rajagopal R., Soumittra N. (2017). Association of polymorphisms in the intron of TCF4 gene to late-onset Fuchs endothelial corneal dystrophy: an Indian cohort study. Indian J. Ophthalmol..

[bib188] Reddy K., Jenquin J.R., Cleary J.D., Berglund J.A. (2019). Mitigating RNA toxicity in myotonic dystrophy using small molecules. Int. J. Mol. Sci..

[bib189] Reed N.S., Deal J.A., Huddle M.G., Betz J.F., Bailey B.E., McGlumphy E.J., Eghrari A.O., Riazuddin S.A., Lin F.R., Gottsch J.D. (2018). Pilot study of audiometric patterns in fuchs corneal dystrophy. JSLHR (J. Speech Lang. Hear. Res.).

[bib190] Renton A.E., Majounie E., Waite A., Simon-Sanchez J., Rollinson S., Gibbs J.R., Schymick J.C., Laaksovirta H., van Swieten J.C., Myllykangas L., Kalimo H., Paetau A., Abramzon Y., Remes A.M., Kaganovich A., Scholz S.W., Duckworth J., Ding J., Harmer D.W., Hernandez D.G., Johnson J.O., Mok K., Ryten M., Trabzuni D., Guerreiro R.J., Orrell R.W., Neal J., Murray A., Pearson J., Jansen I.E., Sondervan D., Seelaar H., Blake D., Young K., Halliwell N., Callister J.B., Toulson G., Richardson A., Gerhard A., Snowden J., Mann D., Neary D., Nalls M.A., Peuralinna T., Jansson L., Isoviita V.M., Kaivorinne A.L., Holtta-Vuori M., Ikonen E., Sulkava R., Benatar M., Wuu J., Chio A., Restagno G., Borghero G., Sabatelli M., Consortium I., Heckerman D., Rogaeva E., Zinman L., Rothstein J.D., Sendtner M., Drepper C., Eichler E.E., Alkan C., Abdullaev Z., Pack S.D., Dutra A., Pak E., Hardy J., Singleton A., Williams N.M., Heutink P., Pickering-Brown S., Morris H.R., Tienari P.J., Traynor B.J. (2011). A hexanucleotide repeat expansion in C9ORF72 is the cause of chromosome 9p21-linked ALS-FTD. Neuron.

[bib191] Riazuddin S.A., McGlumphy E.J., Yeo W.S., Wang J., Katsanis N., Gottsch J.D. (2011). Replication of the TCF4 intronic variant in late-onset Fuchs corneal dystrophy and evidence of independence from the FCD2 locus. Invest. Ophthalmol. Vis. Sci..

[bib192] Riazuddin S.A., Parker D.S., McGlumphy E.J., Oh E.C., Iliff B.W., Schmedt T., Jurkunas U., Schleif R., Katsanis N., Gottsch J.D. (2012). Mutations in LOXHD1, a recessive-deafness locus, cause dominant late-onset Fuchs corneal dystrophy. Am. J. Hum. Genet..

[bib193] Riazuddin S.A., Vasanth S., Katsanis N., Gottsch J.D. (2013). Mutations in AGBL1 cause dominant late-onset Fuchs corneal dystrophy and alter protein-protein interaction with TCF4. Am. J. Hum. Genet..

[bib194] Riazuddin S.A., Vithana E.N., Seet L.F., Liu Y., Al-Saif A., Koh L.W., Heng Y.M., Aung T., Meadows D.N., Eghrari A.O., Gottsch J.D., Katsanis N. (2010). Missense mutations in the sodium borate cotransporter SLC4A11 cause late-onset Fuchs corneal dystrophy. Hum. Mutat..

[bib195] Riazuddin S.A., Zaghloul N.A., Al-Saif A., Davey L., Diplas B.H., Meadows D.N., Eghrari A.O., Minear M.A., Li Y.J., Klintworth G.K., Afshari N., Gregory S.G., Gottsch J.D., Katsanis N. (2010). Missense mutations in TCF8 cause late-onset Fuchs corneal dystrophy and interact with FCD4 on chromosome 9p. Am. J. Hum. Genet..

[bib196] Rodriguez C.M., Todd P.K. (2019). New pathologic mechanisms in nucleotide repeat expansion disorders. Neurobiol. Dis..

[bib197] Rong Z., Hu J., Corey D.R., Mootha V.V. (2019). Quantitative studies of muscleblind proteins and their interaction with TCF4 RNA foci support involvement in the mechanism of fuchs' dystrophy. Invest. Ophthalmol. Vis. Sci..

[bib198] Roussos P. (2012). Transcription factor 4 as an important determinant of gating function in schizophrenia. Proc. Natl. Acad. Sci. U. S. A..

[bib199] Roy O., Leclerc V.B., Bourget J.M., Theriault M., Proulx S. (2015). Understanding the process of corneal endothelial morphological change in vitro. Invest. Ophthalmol. Vis. Sci..

[bib200] Rzuczek S.G., Colgan L.A., Nakai Y., Cameron M.D., Furling D., Yasuda R., Disney M.D. (2017). Precise small-molecule recognition of a toxic CUG RNA repeat expansion. Nat. Chem. Biol..

[bib201] Santo R.M., Yamaguchi T., Kanai A., Okisaka S., Nakajima A. (1995). Clinical and histopathologic features of corneal dystrophies in Japan. Ophthalmology.

[bib202] Santoro M., Masciullo M., Silvestri G., Novelli G., Botta A. (2017). Myotonic dystrophy type 1: role of CCG, CTC and CGG interruptions within DMPK alleles in the pathogenesis and molecular diagnosis. Clin. Genet..

[bib203] Sepp M., Kannike K., Eesmaa A., Urb M., Timmusk T. (2011). Functional diversity of human basic helix-loop-helix transcription factor TCF4 isoforms generated by alternative 5' exon usage and splicing. PloS One.

[bib204] Seriola A., Spits C., Simard J.P., Hilven P., Haentjens P., Pearson C.E., Sermon K. (2011). Huntington's and myotonic dystrophy hESCs: down-regulated trinucleotide repeat instability and mismatch repair machinery expression upon differentiation. Hum. Mol. Genet..

[bib205] Skerjanc I.S., Truong J., Filion P., McBurney M.W. (1996). A splice variant of the ITF-2 transcript encodes a transcription factor that inhibits MyoD activity. J. Biol. Chem..

[bib206] Skorodumova L.O., Belodedova A.V., Antonova O.P., Sharova E.I., Akopian T.A., Selezneva O.V., Kostryukova E.S., Malyugin B.E. (2018). CTG18.1 expansion is the best classifier of late-onset fuchs' corneal dystrophy among 10 biomarkers in a cohort from the European part of Russia. Invest. Ophthalmol. Vis. Sci..

[bib207] Slean M.M., Panigrahi G.B., Ranum L.P., Pearson C.E. (2008). Mutagenic roles of DNA "repair" proteins in antibody diversity and disease-associated trinucleotide repeat instability. DNA Repair.

[bib208] Sobczak K., Wheeler T.M., Wang W., Thornton C.A. (2013). RNA interference targeting CUG repeats in a mouse model of myotonic dystrophy. Mol. Ther..

[bib209] Sobrado V.R., Moreno-Bueno G., Cubillo E., Holt L.J., Nieto M.A., Portillo F., Cano A. (2009). The class I bHLH factors E2-2A and E2-2B regulate EMT. J. Cell Sci..

[bib210] Soh Y.Q., Kocaba V., Pinto M., Mehta J.S. (2020). Fuchs endothelial corneal dystrophy and corneal endothelial diseases: East meets West. Eye.

[bib211] Soh Y.Q., Peh Swee Lim G., Htoon H.M., Gong X., Mootha V.V., Vithana E.N., Kocaba V., Mehta J.S. (2019). Trinucleotide repeat expansion length as a predictor of the clinical progression of Fuchs' Endothelial Corneal Dystrophy. PloS One.

[bib212] Solberg Y., Rosner M., Belkin M. (1998). The association between cigarette smoking and ocular diseases. Surv. Ophthalmol..

[bib213] Soliman A.Z., Xing C., Radwan S.H., Gong X., Mootha V.V. (2015). Correlation of severity of fuchs endothelial corneal dystrophy with triplet repeat expansion in TCF4. JAMA Ophthalmol.

[bib214] Song G., Napoli E., Wong S., Hagerman R., Liu S., Tassone F., Giulivi C. (2016). Altered redox mitochondrial biology in the neurodegenerative disorder fragile X-tremor/ataxia syndrome: use of antioxidants in precision medicine. Mol Med (Cambridge, Mass.).

[bib215] Soragni E., Petrosyan L., Rinkoski T.A., Wieben E.D., Baratz K.H., Fautsch M.P., Gottesfeld J.M. (2018). Repeat-associated non-ATG (RAN) translation in fuchs' endothelial corneal dystrophy. Invest. Ophthalmol. Vis. Sci..

[bib216] Stadtmauer E.A., Fraietta J.A., Davis M.M., Cohen A.D., Weber K.L., Lancaster E., Mangan P.A., Kulikovskaya I., Gupta M., Chen F., Tian L., Gonzalez V.E., Xu J., Jung I.Y., Melenhorst J.J., Plesa G., Shea J., Matlawski T., Cervini A., Gaymon A.L., Desjardins S., Lamontagne A., Salas-Mckee J., Fesnak A., Siegel D.L., Levine B.L., Jadlowsky J.K., Young R.M., Chew A., Hwang W.T., Hexner E.O., Carreno B.M., Nobles C.L., Bushman F.D., Parker K.R., Qi Y., Satpathy A.T., Chang H.Y., Zhao Y., Lacey S.F., June C.H. (2020). CRISPR-engineered T cells in patients with refractory cancer. Science.

[bib217] Stehouwer M., Bijlsma W.R., Van der Lelij A. (2011). Hearing disability in patients with Fuchs' endothelial corneal dystrophy: unrecognized co-pathology?. Clin. Ophthalmol..

[bib218] Stucki D.M., Ruegsegger C., Steiner S., Radecke J., Murphy M.P., Zuber B., Saxena S. (2016). Mitochondrial impairments contribute to Spinocerebellar ataxia type 1 progression and can be ameliorated by the mitochondria-targeted antioxidant MitoQ. Free Rad BioMed.

[bib219] Sun S.Y., Wacker K., Baratz K.H., Patel S.V. (2019). Determining subclinical edema in fuchs endothelial corneal dystrophy: revised classification using Scheimpflug tomography for preoperative assessment. Ophthalmology.

[bib220] Sundin O.H., Jun A.S., Broman K.W., Liu S.H., Sheehan S.E., Vito E.C., Stark W.J., Gottsch J.D. (2006). Linkage of late-onset Fuchs corneal dystrophy to a novel locus at 13pTel-13q12.13. Invest. Ophthalmol. Vis. Sci..

[bib221] Sweatt J.D. (2013). Pitt-Hopkins Syndrome: intellectual disability due to loss of TCF4-regulated gene transcription. Exp. Mol. Med..

[bib222] Sznajder L.J., Swanson M.S. (2019). Short tandem repeat expansions and RNA-mediated pathogenesis in myotonic dystrophy. Int. J. Mol. Sci..

[bib223] Sznajder L.J., Thomas J.D., Carrell E.M., Reid T., McFarland K.N., Cleary J.D., Oliveira R., Nutter C.A., Bhatt K., Sobczak K., Ashizawa T., Thornton C.A., Ranum L.P.W., Swanson M.S. (2018). Intron retention induced by microsatellite expansions as a disease biomarker. Proc. Natl. Acad. Sci. U. S. A..

[bib224] Thalamuthu A., Khor C.C., Venkataraman D., Koh L.W., Tan D.T., Aung T., Mehta J.S., Vithana E.N. (2011). Association of TCF4 gene polymorphisms with Fuchs' corneal dystrophy in the Chinese. Invest. Ophthalmol. Vis. Sci..

[bib225] Thompson R.W., Price M.O., Bowers P.J., Price F.W. (2003). Long-term graft survival after penetrating keratoplasty. Ophthalmology.

[bib226] Todd P.K., Oh S.Y., Krans A., Pandey U.B., Di Prospero N.A., Min K.T., Taylor J.P., Paulson H.L. (2010). Histone deacetylases suppress CGG repeat-induced neurodegeneration via transcriptional silencing in models of fragile X tremor ataxia syndrome. PLoS Genet..

[bib227] Trang H., Stanley S.Y., Thorner P., Faghfoury H., Schulze A., Hawkins C., Pearson C.E., Yoon G. (2015). Massive CAG repeat expansion and somatic instability in maternally transmitted infantile spinocerebellar ataxia type 7. JAMA Neurol.

[bib228] Treangen T.J., Salzberg S.L. (2011). Repetitive DNA and next-generation sequencing: computational challenges and solutions. Nat. Rev. Genet..

[bib229] Tsai Y.C., Greenberg D., Powell J., Hoijer I., Ameur A., Strahl M., al e. (2017). Amplification-free, CRISPR-Cas9 Targeted Enrichment and SMRT Sequencing of Repeat-Expansion Disease Causative Genomic Regions.

[bib230] van der Meulen I.J., Patel S.V., Lapid-Gortzak R., Nieuwendaal C.P., McLaren J.W., van den Berg T.J. (2011). Quality of vision in patients with fuchs endothelial dystrophy and after descemet stripping endothelial keratoplasty. Arch. Ophthalmol..

[bib231] Vasanth S., Eghrari A.O., Gapsis B.C., Wang J., Haller N.F., Stark W.J., Katsanis N., Riazuddin S.A., Gottsch J.D. (2015). Expansion of CTG18.1 trinucleotide repeat in TCF4 is a potent driver of fuchs' corneal dystrophy. Invest. Ophthalmol. Vis. Sci..

[bib232] Vithana E.N., Morgan P.E., Ramprasad V., Tan D.T., Yong V.H., Venkataraman D., Venkatraman A., Yam G.H., Nagasamy S., Law R.W., Rajagopal R., Pang C.P., Kumaramanickevel G., Casey J.R., Aung T. (2008). SLC4A11 mutations in Fuchs endothelial corneal dystrophy. Hum. Mol. Genet..

[bib233] Wagoner M.D., Bohrer L.R., Aldrich B.T., Greiner M.A., Mullins R.F., Worthington K.S., Tucker B.A., Wiley L.A. (2018). Feeder-free differentiation of cells exhibiting characteristics of corneal endothelium from human induced pluripotent stem cells. Biol Open.

[bib234] Wang E.T., Cody N.A., Jog S., Biancolella M., Wang T.T., Treacy D.J., Luo S., Schroth G.P., Housman D.E., Reddy S., Lecuyer E., Burge C.B. (2012). Transcriptome-wide regulation of pre-mRNA splicing and mRNA localization by muscleblind proteins. Cell.

[bib235] Wang Z., Handa J.T., Green W.R., Stark W.J., Weinberg R.S., Jun A.S. (2007). Advanced glycation end products and receptors in Fuchs' dystrophy corneas undergoing Descemet's stripping with endothelial keratoplasty. Ophthalmology.

[bib236] Warner J.P., Barron L.H., Goudie D., Kelly K., Dow D., Fitzpatrick D.R., Brock D.J. (1996). A general method for the detection of large CAG repeat expansions by fluorescent PCR. J. Med. Genet..

[bib237] Wieben E.D., Aleff R.A., Basu S., Sarangi V., Bowman B., McLaughlin I.J., Mills J.R., Butz M.L., Highsmith E.W., Ida C.M., Ekholm J.M., Baratz K.H., Fautsch M.P. (2019). Amplification-free long-read sequencing of TCF4 expanded trinucleotide repeats in Fuchs Endothelial Corneal Dystrophy. PloS One.

[bib238] Wieben E.D., Aleff R.A., Tang X., Butz M.L., Kalari K.R., Highsmith E.W., Jen J., Vasmatzis G., Patel S.V., Maguire L.J., Baratz K.H., Fautsch M.P. (2017). Trinucleotide repeat expansion in the transcription factor 4 (TCF4) gene leads to widespread mRNA splicing changes in fuchs' endothelial corneal dystrophy. Invest. Ophthalmol. Vis. Sci..

[bib239] Wieben E.D., Aleff R.A., Tang X., Kalari K.R., Maguire L.J., Patel S.V., Baratz K.H., Fautsch M.P. (2018). Gene expression in the corneal endothelium of Fuchs endothelial corneal dystrophy patients with and without expansion of a trinucleotide repeat in TCF4. PloS One.

[bib240] Wieben E.D., Aleff R.A., Tosakulwong N., Butz M.L., Highsmith W.E., Edwards A.O., Baratz K.H. (2012). A common trinucleotide repeat expansion within the transcription factor 4 (TCF4, E2-2) gene predicts Fuchs corneal dystrophy. PloS One.

[bib241] Wieben E.D., Baratz K.H., Aleff R.A., Kalari K.R., Tang X., Maguire L.J., Patel S.V., Fautsch M.P. (2019). Gene expression and missplicing in the corneal endothelium of patients with a TCF4 trinucleotide repeat expansion without fuchs' endothelial corneal dystrophy. Invest. Ophthalmol. Vis. Sci..

[bib242] Winkler N.S., Milone M., Martinez-Thompson J.M., Raja H., Aleff R.A., Patel S.V., Fautsch M.P., Wieben E.D., Baratz K.H. (2018). Fuchs' endothelial corneal dystrophy in patients with myotonic dystrophy, type 1. Invest. Ophthalmol. Vis. Sci..

[bib243] Wohrle D., Kennerknecht I., Wolf M., Enders H., Schwemmle S., Steinbach P. (1995). Heterogeneity of DM kinase repeat expansion in different fetal tissues and further expansion during cell proliferation in vitro: evidence for a casual involvement of methyl-directed DNA mismatch repair in triplet repeat stability. Hum. Mol. Genet..

[bib244] Wright A.F., Dhillon B. (2010). Major progress in Fuchs's corneal dystrophy. N. Engl. J. Med..

[bib245] Xia H., Jahr F.M., Kim N.K., Xie L., Shabalin A.A., Bryois J., Sweet D.H., Kronfol M.M., Palasuberniam P., McRae M., Riley B.P., Sullivan P.F., van den Oord E.J., McClay J.L. (2018). Building a schizophrenia genetic network: transcription factor 4 regulates genes involved in neuronal development and schizophrenia risk. Hum. Mol. Genet..

[bib246] Xing C., Gong X., Hussain I., Khor C.C., Tan D.T., Aung T., Mehta J.S., Vithana E.N., Mootha V.V. (2014). Transethnic replication of association of CTG18.1 repeat expansion of TCF4 gene with Fuchs' corneal dystrophy in Chinese implies common causal variant. Invest. Ophthalmol. Vis. Sci..

[bib247] Yadava R.S., Kim Y.K., Mandal M., Mahadevan K., Gladman J.T., Yu Q., Mahadevan M.S. (2019). MBNL1 overexpression is not sufficient to rescue the phenotypes in a mouse model of RNA toxicity. Hum. Mol. Genet..

[bib248] Yoon S.O., Chikaraishi D.M. (1994). Isolation of two E-box binding factors that interact with the rat tyrosine hydroxylase enhancer. J. Biol. Chem..

[bib249] Zarouchlioti C., Sanchez-Pintado B., Hafford Tear N.J., Klein P., Liskova P., Dulla K., Semo M., Vugler A.A., Muthusamy K., Dudakova L., Levis H.J., Skalicka P., Hysi P., Cheetham M.E., Tuft S.J., Adamson P., Hardcastle A.J., Davidson A.E. (2018). Antisense therapy for a common corneal dystrophy ameliorates TCF4 repeat expansion-mediated toxicity. Am. J. Hum. Genet..

[bib250] Zerbino D.R., Achuthan P., Akanni W., Amode M.R., Barrell D., Bhai J., Billis K., Cummins C., Gall A., Giron C.G., Gil L., Gordon L., Haggerty L., Haskell E., Hourlier T., Izuogu O.G., Janacek S.H., Juettemann T., To J.K., Laird M.R., Lavidas I., Liu Z., Loveland J.E., Maurel T., McLaren W., Moore B., Mudge J., Murphy D.N., Newman V., Nuhn M., Ogeh D., Ong C.K., Parker A., Patricio M., Riat H.S., Schuilenburg H., Sheppard D., Sparrow H., Taylor K., Thormann A., Vullo A., Walts B., Zadissa A., Frankish A., Hunt S.E., Kostadima M., Langridge N., Martin F.J., Muffato M., Perry E., Ruffier M., Staines D.M., Trevanion S.J., Aken B.L., Cunningham F., Yates A., Flicek P. (2018). Ensembl 2018. Nucleic Acids Res..

[bib251] Zhang J., McGhee C.N.J., Patel D.V. (2019). The molecular basis of fuchs' endothelial corneal dystrophy. Mol. Diagn. Ther..

[bib252] Zhang K., Donnelly C.J., Haeusler A.R., Grima J.C., Machamer J.B., Steinwald P., Daley E.L., Miller S.J., Cunningham K.M., Vidensky S., Gupta S., Thomas M.A., Hong I., Chiu S.L., Huganir R.L., Ostrow L.W., Matunis M.J., Wang J., Sattler R., Lloyd T.E., Rothstein J.D. (2015). The C9orf72 repeat expansion disrupts nucleocytoplasmic transport. Nature.

[bib253] Zhang K., Pang K., Wu X. (2014). Isolation and transplantation of corneal endothelial cell-like cells derived from in-vitro-differentiated human embryonic stem cells. Stem Cell. Dev..

[bib254] Zinflou C., Rochette P.J. (2017). Ultraviolet A-induced oxidation in cornea: characterization of the early oxidation-related events. Free Radic. Biol. Med..

[bib255] Zoega G.M., Fujisawa A., Sasaki H., Kubota A., Sasaki K., Kitagawa K., Jonasson F. (2006). Prevalence and risk factors for cornea guttata in the Reykjavik Eye Study. Ophthalmology.

[bib256] Zu T., Cleary J.D., Liu Y., Banez-Coronel M., Bubenik J.L., Ayhan F., Ashizawa T., Xia G., Clark H.B., Yachnis A.T., Swanson M.S., Ranum L.P.W. (2017). RAN translation regulated by muscleblind proteins in myotonic dystrophy type 2. Neuron.

[bib257] Zu T., Gibbens B., Doty N.S., Gomes-Pereira M., Huguet A., Stone M.D., Margolis J., Peterson M., Markowski T.W., Ingram M.A., Nan Z., Forster C., Low W.C., Schoser B., Somia N.V., Clark H.B., Schmechel S., Bitterman P.B., Gourdon G., Swanson M.S., Moseley M., Ranum L.P. (2011). Non-ATG-initiated translation directed by microsatellite expansions. Proc. Natl. Acad. Sci. U. S. A..

[bib258] Zu T., Liu Y., Banez-Coronel M., Reid T., Pletnikova O., Lewis J., Miller T.M., Harms M.B., Falchook A.E., Subramony S.H., Ostrow L.W., Rothstein J.D., Troncoso J.C., Ranum L.P. (2013). RAN proteins and RNA foci from antisense transcripts in C9ORF72 ALS and frontotemporal dementia. Proc. Natl. Acad. Sci. U. S. A..

